# Age-dependent progression from clearance to vulnerability in the early response of periventricular microglia to α-synuclein toxic species

**DOI:** 10.1186/s13024-025-00816-1

**Published:** 2025-03-05

**Authors:** Mª Salomé Sirerol-Piquer, Ana Perez-Villalba, Pere Duart-Abadia, Germán Belenguer, Ulises Gómez-Pinedo, Laura Blasco-Chamarro, Pau Carrillo-Barberà, Azucena Pérez-Cañamás, Victoria Navarro-Garrido, Benjamin Dehay, Miquel Vila, Javier Vitorica, Francisco Pérez-Sánchez, Isabel Fariñas

**Affiliations:** 1 Centro de Investigación Biomédica en Red de Enfermedades Neurodegenerativas (CIBERNED), Madrid, Spain; 2 Departamento de Biología Celular, Biología Funcional y Antropología Física, Universidad de Valencia, Burjassot, Spain; 3 Instituto de Biotecnología y Biomedicina (BioTecMed), Universidad de Valencia, Burjassot, Spain; 4 Laboratory of Neurobiology, Institute of Neurosciences, Hospital Clínico San Carlos Health Research Institute, Universidad Complutense de Madrid, Madrid, Spain; 5 Instituto de Biomedicina de Sevilla (IBiS), Universidad de Sevilla, Seville, Spain; 6 Departamento Bioquímica y Biología Molecular, Universidad de Sevilla, Seville, Spain; 7 Univ. Bordeaux, CNRS, IMN, UMR 5293, Bordeaux, F-33000 France; 8 Neurodegenerative Diseases Research Group, Vall d´Hebron Research Institute, Autonomous University of Barcelona, Barcelona, Spain; 9 Catalan Institution for Research and Advanced Studies (ICREA), Barcelona, Spain; 10 Present Address: L.A.B.P. (Laboratory of Animal Behavior Phenotype), Facultad de Psicología. UCV, Valencia, Spain

**Keywords:** Alpha-synuclein, Microglia, Aging, Parkinson’s disease, Lewy bodies, PFFs, CSF

## Abstract

Cytoplasmic alpha-synuclein (αSyn) aggregates are a typical feature of Parkinson’s disease (PD). Extracellular insoluble αSyn can induce pathology in healthy neurons suggesting that PD neurodegeneration may spread through cell-to-cell transfer of αSyn proteopathic seeds. Early pro-homeostatic reaction of microglia to toxic forms of αSyn remains elusive, which is especially relevant considering the recently uncovered microglial molecular diversity. Here, we show that periventricular microglia of the subependymal neurogenic niche monitor the cerebrospinal fluid and can rapidly phagocytize and degrade different aggregated forms of αSyn delivered into the lateral ventricle. However, this clearing ability worsens with age, leading to an increase in microglia with aggregates in aged treated mice, an accumulation also observed in human PD samples. We also show that exposure of aged microglia to aggregated αSyn isolated from human PD samples results in the phosphorylation of the endogenous protein and the generation of αSyn seeds that can transmit the pathology to healthy neurons. Our data indicate that while microglial phagocytosis rapidly clears toxic αSyn, aged microglia can contribute to synucleinopathy spreading.

**Supplementary Information:**

The online version contains supplementary material available at 10.1186/s13024-025-00816-1.

## Introduction

Parkinson’s disease (PD) is characterized by abnormal intraneuronal aggregates of the synaptic protein alpha-synuclein (αSyn) known as Lewy bodies (LB) [1–3]. Mounting evidence sustains the idea that aggregated pathological αSyn may reach the extracellular milieu and induce the trans-cellular spreading of the pathology. αSyn deposits spanning progressively larger brain areas in *postmortem* PD samples can be directly associated with the course of the disease, and embryonic mesencephalic neurons grafted in the striatum of PD patients can develop LB pathology [4–7]. Injection of LB fractions into the brain parenchyma of mice and primates results in the long-term pathological modification and aggregation of endogenous αSyn into LB-like lesions [8–11]. In vitro-aggregated forms of recombinant monomeric αSyn, consisting of oligomers, ribbons, or pre-formed fibrils (PFFs), can also induce the seeding of αSyn pathology in neuronal cultures. Furthermore, toxic αSyn oligomer-containing exosomes from affected neurons reportedly mediate the spreading of the pathology to other neurons when injected in vivo [12–23]. Administration of these different αSyn toxic forms has, therefore, emerged as a promising modeling strategy for the study of pathological αSyn spreading, as it enables spatiotemporal studies of the induction of the disease and its progression [8, 20, 24–26].

Although cell-to-cell transmission of pathologic αSyn between interconnected brain regions has suggested prion-like effects, the spread pattern does not necessarily adhere to neural connectivity [24, 27]. In this context, the cerebrospinal fluid (CSF) has recently emerged as a potential brain reservoir of misfolded αSyn [28, 29]. Indeed, pathogenic aggregated αSyn found in exosomes isolated from the CSF of PD patients can initiate the oligomerization of soluble αSyn in cells [23, 30, 31]. The mammalian brain ventricles, where the CSF that bathes the central nervous system is generated, are lined up by a monolayer of ependymocytes that act as a physical barrier between the fluid and the brain parenchyma [32]. At the level of the lateral ventricles (LV), the lining wall includes the germinal niche known as the subependymal zone (SEZ), where glial fibrillary acidic protein (GFAP)-positive neural stem cells (NSCs) continuously produce new neurons for olfactory circuits. The SEZ includes the NSCs and their progeny and other cellular elements, such as niche astrocytes, vascular elements, axonal projections, and microglial cells [32]. Microglia are highly dynamic brain-resident innate immune cells that continuously survey their surrounding microenvironment to maintain homeostasis, mainly through the phagocytosis of pathogens, dead cells, and debris [33]. Across most adult brain regions, Tmem119-positive homeostatic microglial cells appear remarkably homogeneous at the molecular level. In contrast, developmental Tmem119-negative microglia are much more heterogeneous. These cells are found in different areas during fetal and postnatal development, but in the adult brain, they become restricted to regions of persistent neurogenesis, i.e., the SEZ [34, 35]. Intriguingly, these proliferative-region-associated microglia (PAM) exhibit gene expression signatures previously associated with the degenerative disease-associated microglia (DAM) found associated with Aβ-plaques and other abnormal proteinaceous deposits [36]. Although brain microglial cells are generally thought to participate in the phagocytosis and clearance of αSyn [37], the potential response of periventricular non-homeostatic microglia to aggregated CSF-derived αSyn remains elusive. Furthermore, the SEZ location provides an ideal scenario to test the early responses of cells to toxic proteins delivered into the CSF without the confounding tissue injury reaction that characterizes intraparenchymal injections.

Here, we show that subependymal periventricular microglia actively survey the CSF by extending projections that cross the ependymal layer and are highly efficient in clearing both αSyn PFFs and LB-enriched fractions containing αSyn, but their phagocytic/degrading function to remove these toxic forms of αSyn is reduced with age. Compromised clearance of αSyn LB-fractions with age results in the spreading of the pathology to other microglial cells and neurons. Our results indicate that periventricular microglia constitute a first-line defense to avoid the spreading of toxic forms of αSyn from the CSF, but can contribute to the disease progression in the aging brain.

## Results

### Periventricular microglia survey the lateral ventricles and phagocytize aggregated αSyn from the CSF

**Fig. 1:**
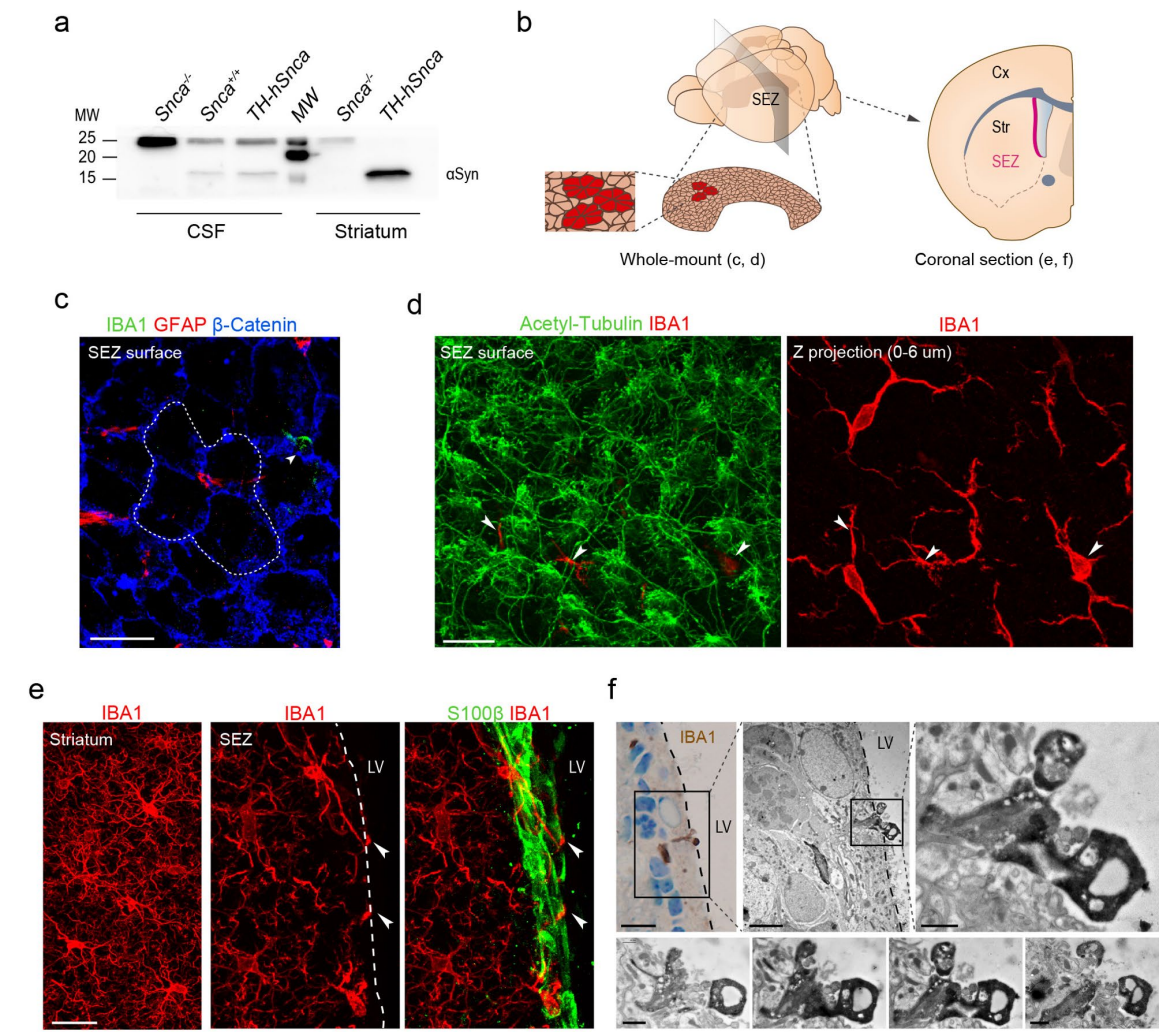
Periventricular microglia have access to αSyn from the CSF. **(a)** Western blot showing the presence of αSyn in the CSF of *Snca*^+/+^ and TH-*hSnca*, but not *Snca*^−/−^ mice; detection in the striatum is shown as a control. **(b)** Schematic representation illustrating the SEZ location in a mouse brain, a whole-mount-en-face preparation and a coronal section containing the SEZ. Cx: cortex, Str: striatum, LV: lateral ventricle. **(c)** Confocal image of a whole-mount-en-face preparation of a dissected SEZ immunostained for microglia (IBA1, green), GFAP (red) and β-catenin (blue). The dashed white line delineates the rosette of ependymocytes surrounding a few GFAP^+^ NSC cell apical processes. **(d)** Left panel: Confocal image of the ventricular surface of a SEZ whole-mount-en-face preparation stained for acetylated-tubulin (green), a marker of ependymal cilia, and IBA1 (red) showing cytoplasmic processes of microglial cells contacting the LV through the ependymal layer (pointed by arrowheads). Right panel: Z-stack of confocal images from the ependymal surface (0–6 μm) showing the cell bodies of microglial cells that are the origin of the processes shown in the left panel. **(e)** Confocal images of coronal sections displaying microglia (IBA1, red) in the striatum (left panel) and the SEZ (middle panel). Right panel: confocal images of microglia (red) contacting the LV through the ependymal layer stained for S100β (green). **(f)** Light microscopy picture of a toluidine blue**-**stained 1-µm-thick section and EM micrographs showing direct contact of IBA1^+^ DAB-reacted microglia with the LV. Bottom: reconstruction of the microglia contacting the ventricle. Scale bars: c-e, 20 μm; f, 10 μm (light microscopy), 4 μm (EM, low magnification), and 1 μm (EM, high magnification)

We first addressed the possibility that neuron-derived αSyn could be naturally present in the CSF of mouse brains. Detection of αSyn by Western blot in CSF samples extracted from the cisterna magna of 2-month-old wild-type and TH-hαSyn transgenic mice expressing human αSyn only in catecholaminergic neurons [38] revealed an αSyn specific band that could not be seen in CSF obtained from *Snca* null (*Snca*^*−/−*^) mice (Fig. 1a; striatal homogenates of the same mice were used as controls). To obtain a quantitative assessment of the CSF levels of αSyn produced by catecholaminergic neurons, we performed an ELISA using antibodies specific to the human protein. CSF and striatal homogenates pooled from three TH-hαSyn mice revealed concentrations of the transgenic αSyn of 0.24 ng/ml (within the range of human CSF concentrations [39]) and 17 ng/ml (see also [40, 41]), respectively. These results confirmed the natural presence of αSyn derived from monoaminergic neurons in the CSF, suggesting that this fluid can be a natural vehicle for the dissemination of neuronal αSyn.

The SEZ neurogenic niche is located in the LV wall immediately adjacent to the lining ependymal layer, and whole-mount preparations of the LV wall allow the visualization of the 3D relationships among its different cell types and with the CSF [42] (Fig. 1b). Confocal 3D microscopy analysis after immunofluorescent detection for cell-specific antigens has revealed that GFAP-positive NSCs intercalate their apical cytoplasmic process perpendicularly among the ependymocytes and access the CSF [42] (see Fig. 1c for an example). Microglia is another conspicuous element in the SEZ and their labeling in whole-mount preparations with antibodies to ionized calcium-binding adaptor molecule 1 (IBA1) revealed IBA1-positive filopodia-like protrusions embedded among ependymal cells and contacting the ventricular space, as seen in confocal 3D projections (Fig. 1c, d). Our analysis in static images indicated 7.44 ± 1.02 × 10^− 4^ microglial projections per µm^2^ contacting the LV. The intercalating protrusions could also be observed in conventional sections and were corroborated by correlative light-electron microscopy (EM) analysis in IBA1-immunoperoxidase reacted samples. Phagocytic protrusions were distinctly observed extending into the LV lumen (Fig. 1e, f). SEZ microglia could also be labeled with antibodies to the purinergic receptor P2RY12 or visualized in heterozygous mice of the *Cx3r1*^*eGFP*^ knock-in reporter strain carrying an enhanced GFP-coding allele in the endogenous *Cx3cr1* locus [43] (Suppl. Figure 1a-c). In addition, some SEZ microglia exhibited specific PAM and DAM traits, such as increased levels of CLEC7A [36] and a characteristic semi-amoeboid shape compared to the ramified one of other brain areas, such as the adjacent striatum (Suppl. Figure 1a-c), that is in line with their apparently higher state of reactivity compared to microglia in non-neurogenic regions [33, 44]. The results indicated that subependymal microglia can play a role in CSF surveillance, likely acting as one of the first lines of defense against toxic components in the CSF.

**Fig. 2:**
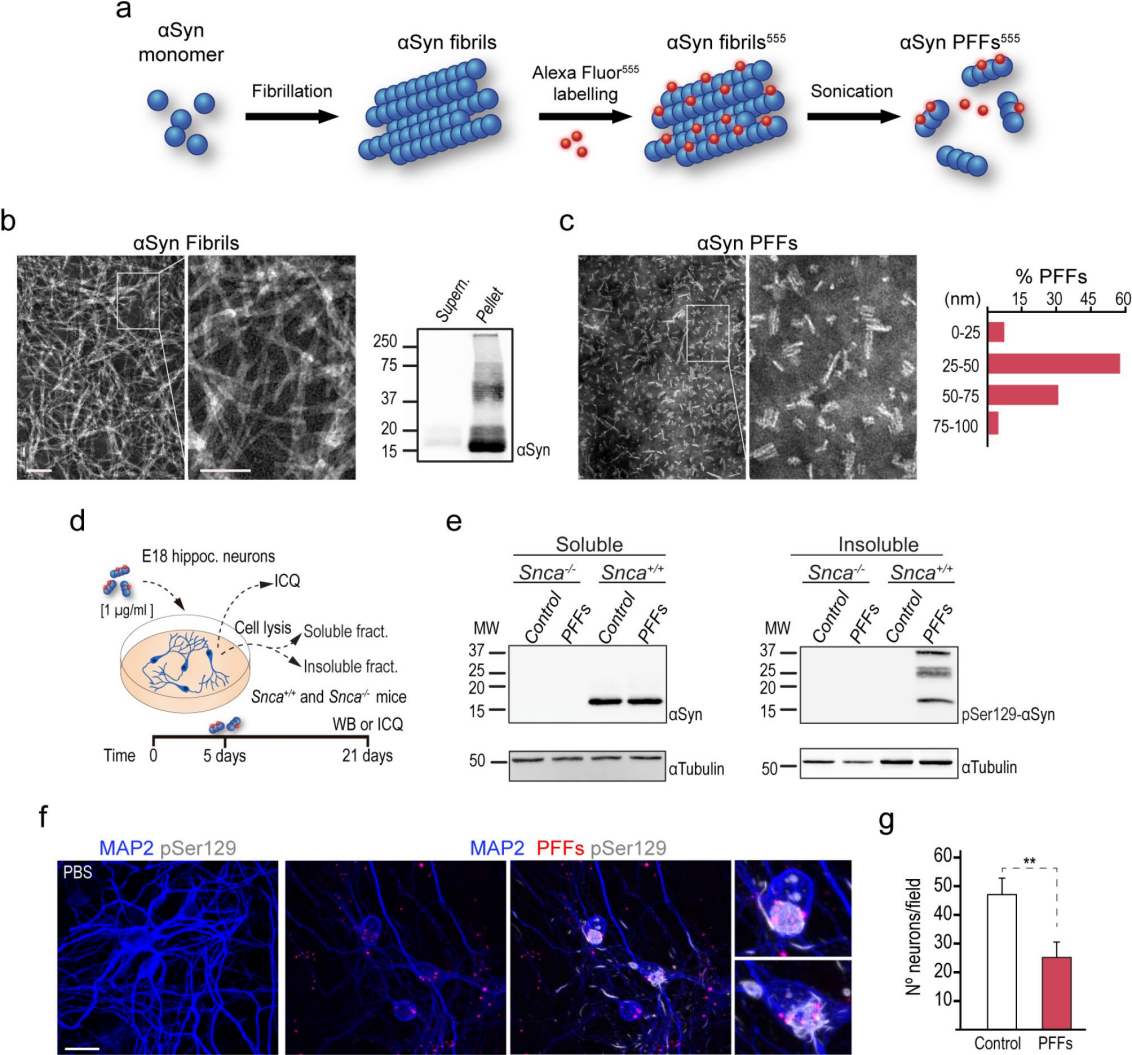
αSyn PFFs production and validation. **(a)** Schematic representation of the protocol for generating αSyn PFFs from monomers (see Material and Methods). **(b)** Fibrillation assessment by EM and Western blot before sonication. **(c)** EM images and size distribution of the αSyn PFFs after sonication. **(d)** Schematic depicting the treatment of E18 hippocampal neurons with PFFs and analysis. **(e)** Detection of endogenous αSyn by Western blot in soluble (left) and insoluble (right) extracts from hippocampal neuron cultures of *Snca*^*+/+*^ and *Snca*^*−/−*^ mice. **(f)** Confocal images of cultures of hippocampal neurons treated with PBS or PFFs (red) and immunostained for MAP2 (blue) and pSer129-αSyn (white). **(g)** Quantification of the number of neurons *per* field in hippocampal neuron cultures treated with PFFs. Scale bars: b and c, left image 200 nm and right image 100 nm; f, 20 μm

Based on our observations, we decided to test the interaction of periventricular microglia with toxic forms of αSyn delivered into the CSF. αSyn pre-formed fibrils (PFFs) were generated in vitro from endotoxin-free recombinant murine αSyn following the standard procedures of the Michael J. Fox Foundation and tagged with an Alexa Fluor 555 fluorophore [24]. The resulting fibrillary assemblies were tested for size and morphology by sedimentation and EM after negative staining (Fig. 2a-c). Subsequently, PFFs were sonicated to obtain assemblies of about 50 nm average size (Fig. 2c) that are reportedly optimal to reproduce αSyn pathology in culture or after injection [24]. Primary hippocampal neurons obtained from *Snca* wild-type and null E18 fetuses were seeded at a density of 50,000 cells *per* cm^2^, treated with 1 µg/ml PFFs or PBS for 5 days, and analyzed 16 days later (Fig. 2d). Analysis by Western blot of the cell soluble and insoluble protein fractions isolated from the treated cultures revealed that the treatment with PFFs resulted in the formation of aggregates of endogenous αSyn in wild-type, that were not detected in *Snca* null cultures. Furthermore, aggregated αSyn carried the pSer129 post-translational modification reportedly associated with toxicity (Fig. 2e). Analysis by immunocytochemistry revealed that most neurons had internalized PFFs (83.68 ± 2.17%; *n* = 3) and were strongly positive for aggregated pSer129-αSyn (Fig. 2f). As a result, overall neuronal viability was compromised in the PFF treated cultures (Fig. 2g). The results indicated that the PFFs generated in vitro could act as seeds and induce endogenous αSyn pathology in neurons, as reported for these types of assemblies [45].

**Fig. 3:**
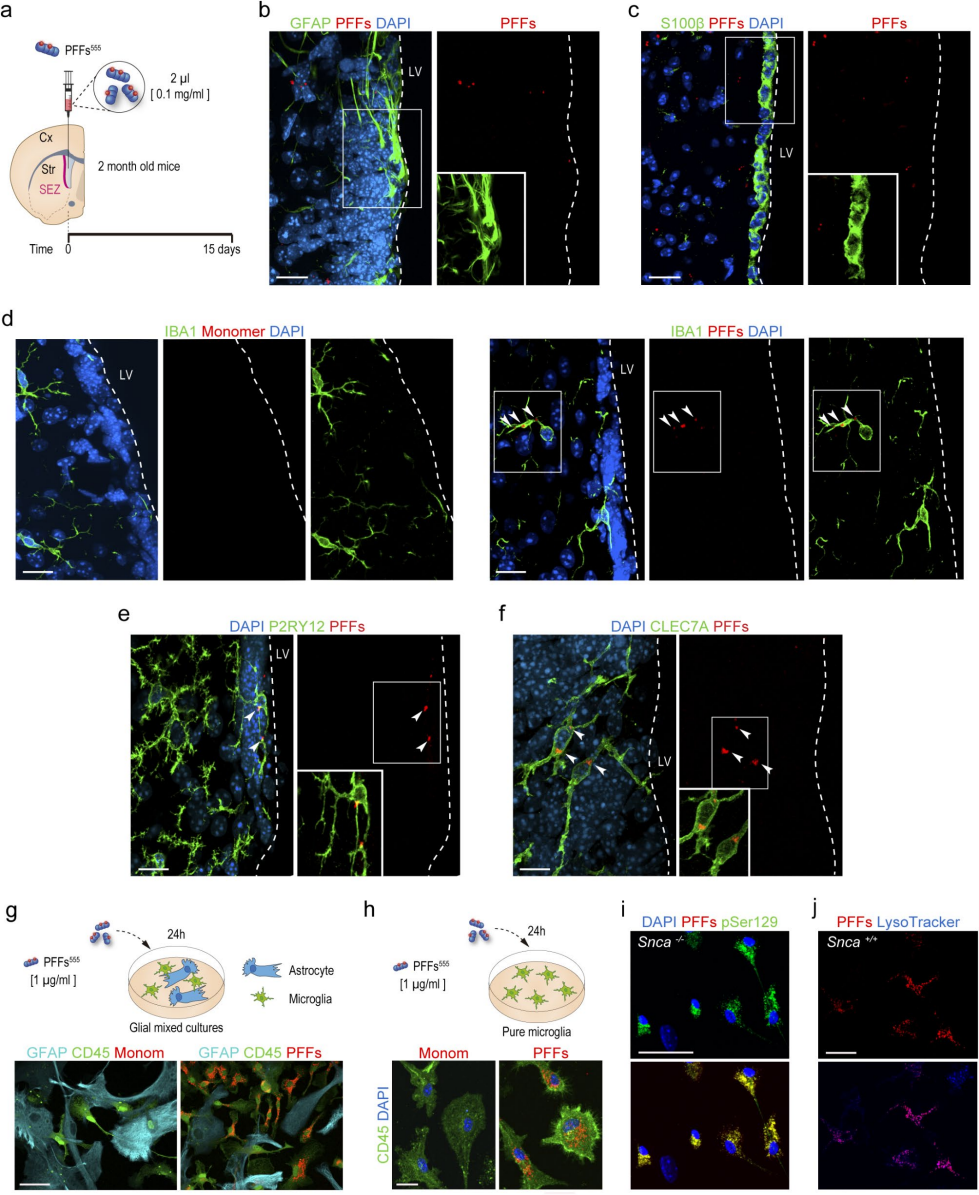
Microglia selectively phagocytize αSyn PFFs from the CSF and culture media. **a.** Schematic representation of the in vivo experiment. **b-f.** Confocal images of the SEZ showing αSyn PFFs (red) 15 days after their infusion into the LV. Absence of PFF uptake in astrocytes (GFAP, green) (**b**) and ependymocytes (S100β, green) (**c**). PFF uptake by microglial cells (green) immunostained for IBA1 (**d**), P2RY12 (**e**), and CLEC7A (**f**). Arrowheads point at PFFs within microglial cells. **g.** Top: schematic representation of the in vitro experiment using mixed glial cell cultures. At the bottom: Detection of αSyn monomer or PFFs (red) in mixed glial cell cultures composed of astrocytes (GFAP, cyan) and microglia (CD45, green). **h.** Top: schematic representation of the in vitro experiment using pure microglia cultures. Bottom: Detection of αSyn monomer or PFFs (red) in pure microglia cultures (CD45, green). **i.** Detection of PFFs (red) and αSyn pSer129 (green) in *Snca*^*−/−*^ mixed glial cultures. **j.** Co-localization of PFFs (red) with lysosomes using LysoTracker (blue). DAPI: blue. Scale bars: b-e 20 μm; g, 40 μm; h, 20 μm; i, 20 μm; j, 30 μm

We next performed stereotaxic injections of 2 µl fluorescently tagged PFFs at 0.1 mg/ml into the LV of 2 month-old mice (Fig. 3a). In contrast to intraparenchymal delivery, injections into the LV do not physically harm the SEZ allowing the evaluation of the short-term response of periventricular microglia without the confounding effects of direct tissue injury. Indeed, we determined the degree of microglial activation 15 days after the injection by measuring the number of IBA1-positive somas and the area occupied by them and found that intracerebroventricular injection of αSyn assemblies did not alter microglial morphology in the SEZ (Suppl. Figure 2a). At this time, PFFs were never found inside S100β-positive ependymocytes that line up the LV and only 3.53 ± 0.73% (*n* = 3) of GFAP-positive astrocytes/NSCs were labeled with PFFs (Fig. 3b, c). In contrast, we could readily detect IBA1-positive, P2RY12-positive, and CLEC7A-positive periventricular microglial cells with PFFs inside (Fig. 3d-f), suggesting the specific involvement of microglia in the clearance from the CSF. We could observe 48.37 ± 2.46% (*n* = 5) of IBA1^+^ and 30.92 ± 4.94% (*n* = 3) of CLEC7A^+^ microglial cells with PFFs inside. The specificity of the PFF uptake was studied in animals injected with PBS or monomeric αSyn as a control, in which we never observed any signal (Fig. 3d). Interestingly, microglial cells with PFFs inside could be observed as early as 2 h after the injection (Suppl. Figure 2b, c) suggesting a very fast phagocytic response. We also used a cytometry-based strategy to quantitate the PFF engulfment by CLEC7A-positive microglia. To do so, we specifically isolated the CLEC7A-positive fraction within the CD11b^+^CD45^low^ microglial population (Suppl. Figure 3a, b) and the cells were incubated with PFFs in suspension for 5 h, washed, and evaluated by flow cytometry. We found that CLEC7A-positive microglia efficiently and rapidly engulfed PFFs (Suppl. Figure 3c). Together, the data indicated that periventricular microglia physiologically survey the CSF and are specifically involved in the clearance of toxic αSyn from the LV.

To confirm the selective uptake of aggregated αSyn by microglia vs. other cells without the spatial constraint of the ependymal barrier, we obtained primary mixed glial cell cultures from the SEZ containing astrocytes and microglial cells and treated them with fluorescent PFFs or monomeric αSyn at 1 µg/ml for 24 h. In agreement with our in vivo analyses, microglial cells were heavily loaded with PFFs. In contrast, GFAP-positive astrocytes did not internalize any αSyn form despite direct exposure (Fig. 3g). In turn, uptake by microglia was not dependent on the presence of astrocytes in the culture, as we could find efficient phagocytosis of PFFs also in pure cultures of brain primary microglia (Fig. 3h). Because other authors have previously observed PFF uptake by astrocytes in vitro [46, 47], these results suggest that the highly efficient phagocytic activity of microglia takes over at young ages. The post-translational pSer129 phosphorylation targets αSyn for degradation by the lysosome [48–50]. We found that PFFs became phosphorylated in S129 after 24 h inside microglial cells in pure cultures that were obtained from *Snca* null animals to eliminate the potentially confounding effect of endogenous αSyn phosphorylation (Fig. 3i; Suppl. Figure 4a). PFF and LysoTracker detection in real-time confocal microscopy revealed that PFF uptake follows the endocytic pathway (Suppl. Video 1). In addition, a Manders’ overlap coefficient M_1_ = 0.973 indicated that virtually all PFFs were in lysosomes 24 h after treatment (Fig. 3j).

### Age-related declines in periventricular microglia capacity reduce PFF clearance from the CSF

**Fig. 4:**
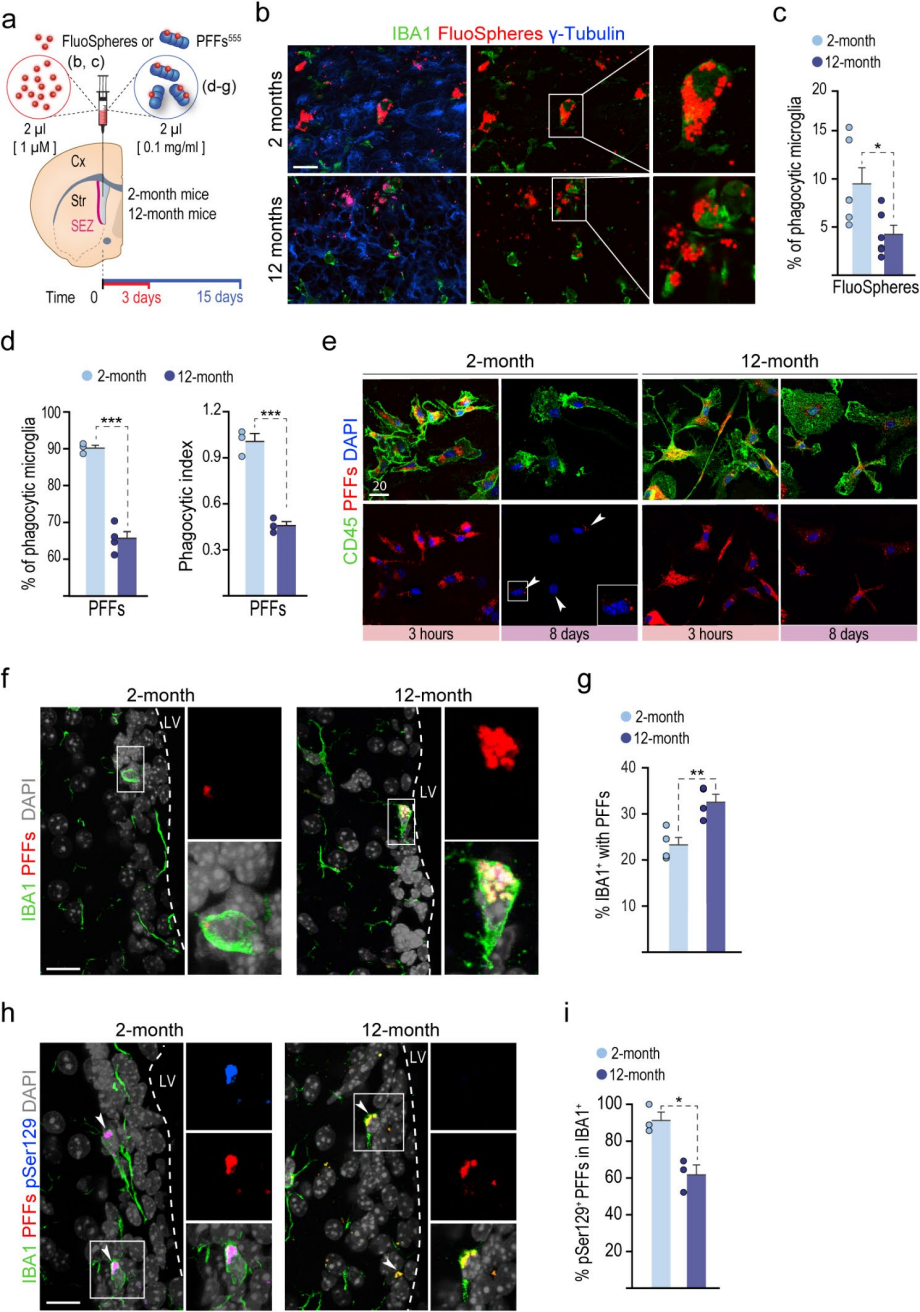
Aging impairs phagocytic and degrading microglial capacity. **(a)** Schematic representation of the injection of red fluorescent microspheres (FluoSpheres, red) or αSyn PFFs (blue) into the right LV of 2- and 12-month-old mice. The animals were euthanized 3 days after the FluoSpheres infusion or 15 days after the infusion of PFFs. **(b)** Whole-mount-en-face preparations of the contralateral dissected SEZ immunostained for γ-tubulin (blue; a marker of cilia centrosomes to show the ependymal surface) and microglia (IBA1, green). **(c)** Percentage of microglia isolated from the ipsilateral SEZ containing FluoSpheres (*n* = 5 mice). Data are presented as mean values ± SEM; Student’s *t-test p* < 0.05. **(d)** Ex vivo phagocytosis assay with αSyn PFFs in microglia isolated from 2- and 12-month-old mice. Percentage of phagocytic microglia and phagocytic index in 2- (*n* = 3) and 12-month-old (*n* = 4) mice. Data are presented as mean values ± SEM; Student’s *t-test p* < 0.001. **(e)** In vitro degradation experiment. Mixed glial cultures obtained from young and aged mice were incubated for 3 h with PFFs (red) and either fixed immediately (3 h) or washed and maintained for 8 days in fresh media (8 days) before fixation and immunostaining for microglia (CD45, green). **(f)** Confocal images of the SEZ of 2- and 12-month-old mice showing PFFs (red) and IBA1^+^ microglia (green) 15 days after infusion. DAPI: grey. **(g)** Quantification of the percentage of microglia containing αSyn PFFs in 2- (*n* = 4) and 12-month-old (*n* = 3) mice. Data are presented as mean values ± SEM; *p* < 0.01, Student’s *t-test*. **(h)** Confocal images of the SEZ of young and aged mice showing PFFs (red), pSer129 (blue) and IBA1^+^ microglia (green) 15 days after infusion. DAPI: grey. **(i)** Quantification of the percentage of microglia containing phosphorylated αSyn PFFs in 2- (*n* = 3) and 12-month-old (*n* = 3) mice. Data are presented as mean values ± SEM; Student’s *t-test*,* p* < 0.05. Scale bars: b, 50 μm; e, 20 μm; f, h 20 μm

Because aging remains the most significant risk factor for developing idiopathic PD, we next decided to evaluate the effect of age on the capacity of periventricular microglia to engulf and degrade αSyn PFFs. In aging mice, microglia reportedly undergo morphological changes, i.e., less ramified cell morphology, reduced process length, increased soma volume, and functional alterations, including impaired phagocytic and lysosomal dysfunction [51–55]. Indeed, immunostainings for the indicator of endosomal/lysosomal activity CD68 in 12- vs. 3-month-old mice revealed an abnormal distribution of the staining in SEZ microglia of elderly mice, suggesting an age-dependent endolysosomal impairment (Suppl. Figure 5a). To functionally evaluate the in vivo phagocytic capacity of SEZ microglia over time, we injected red fluorescent microspheres (FluoSpheres™) into the right LV of 2- and 12-month-old mice (Fig. 4a). Three days after the injection, the ipsilateral SEZs were disaggregated for flow cytometry analysis and the contralateral SEZs were fixed for microscopy analysis. Examination of the whole-mount-en-face preparation of the LV wall by confocal microscopy revealed an apparently reduced proportion of IBA1-positive cells with fluorescent spheres inside and fewer microspheres within each microglial cell in 12-month-old mice (Fig. 4b). In good agreement with these observations, flow cytometry analysis showed that, although the CD11b^+^CD45^low^ microglial fraction recovered from the SEZ of 2-month and 12-month old mice was similar (1.60 ± 0.39 and 1.86 ± 0.4% of all SEZ cells, respectively, *n* = 7), the proportion of fluorescent bead-containing microglial cells was significantly reduced in the samples from aged mice (Fig. 4c).

We next set out to quantitatively test the PFF-specific short-term phagocytic capacity of microglia employing flow cytometry. CD11b^+^CD45^low^ microglia from the brains of 2- and 12-month-old mice were incubated with PFFs in suspension for 5 h, washed, and assayed by flow cytometry. The percentage of phagocytic microglial cells from 12-month-old mice was significantly reduced and they displayed a reduced phagocytic index (Fig. 4d). These data indicated that the phagocytic capacity of periventricular microglia declines over time. We next used an in vitro model to specifically analyze PFF degradation. We performed this experiment in mixed astroglial-microglial cell cultures, as pure microglial cultures from aged mice are extremely difficult to maintain. Cultures obtained from 12-month-old mice and incubated with 1 µg/ml PFFs for 3 h were subsequently washed and fixed to set the initial uptake or washed and maintained alive for 8 days to evaluate clearance. Qualitatively, after 8 days, CD45^+^ microglial cells from 2-month-old mice presented an apparent reduction of PFF content in their cytoplasm, with only small aggregates surrounding the nucleus. In contrast, PFFs were more abundant and appeared more dispersed in the cytoplasm of microglial cells obtained from 12-month-old mice (Fig. 4e). Quantitative analysis showed a significant reduction in the degradative capacity from 82.53 ± 3.82 to 10.54 ± 2.88% (*p* < 0.001) in 2-month and 12-month old mice, respectively.

Our in vitro and ex vivo data indicated that microglia lose phagocytic, but also degradative capacity over time. To evaluate whether the age-related functional decline in microglia capacities could affect the in vivo surveillance of CSF αSyn by microglia, we analyzed the microglial uptake of PFFs injected into the LV of 2- and 12-month-old mice 15 days after infusion (Fig. 4a). The percentage of microglia containing PFFs was significantly increased at 12 months and we could also observe an apparent increase in the PFF load *per* cell (Fig. 4f, g) in line with the reduced degradation capacity observed in vitro. The immunostaining for pSer129 was significantly reduced in 12-month-old mice (Fig. 4h, i), suggesting that a less effective degradation of αSyn assemblies could potentially be due to a less efficient phosphorylation at this age.

We next decided to evaluate in vivo whether a deficient handling at 12 months could result in effects in other cells. As αSyn accumulations are found in astrocytes of autopsy PD samples [56], we paid attention to SEZ GFAP^+^ cells. Interestingly, we found a significantly higher proportion of astrocytes that had up-taken PFFs in 12-month vs. 2 month-old-mice (Suppl. Figure 6a, b). To test the possibility that reduced phagocytosis by microglia could indeed be responsible for the engagement of astrocytes in PFF uptake, we decided to evaluate PFF phagocytosis in knock-in *Cx3cr1*^*eGFP/eGFP*^ young mice. It has been reported that Cx3cr1-deficient microglia from *Cx3cr1*^*eGFP/eGFP*^ young mice exhibit a transcriptome consistent with that of aged Cx3cr1-sufficient animals, suggesting a premature aging transcriptomic signature [57]. In agreement with this, microglial cells isolated from young *Cx3cr1*^*eGFP/eGFP*^ mice and incubated with PFFs in suspension for 5 h evidenced a significantly reduced phagocytic activity, compared to *Cx3cr1*^*+/eGFP*^ or *Cx3cr1*^*+/+*^, as assayed by flow cytometry (Suppl. Figure 6c). In line with this and with the previous results, we observed increased proportions of astrocytes with PFFs inside in SEZ sections of young mice lacking CX3CR1 (Suppl. Figure 6d, e). All these data together indicated that periventricular microglia phagocytize and degrade the PFFs less efficiently at 12 months and, furthermore, that this functional decline leads to the involvement of astrocytes/NSCs in the uptake of PFFs.

### Reduced clearance of LB-derived aggregated αSyn from the LV results in modification of the endogenous protein

**Fig. 5:**
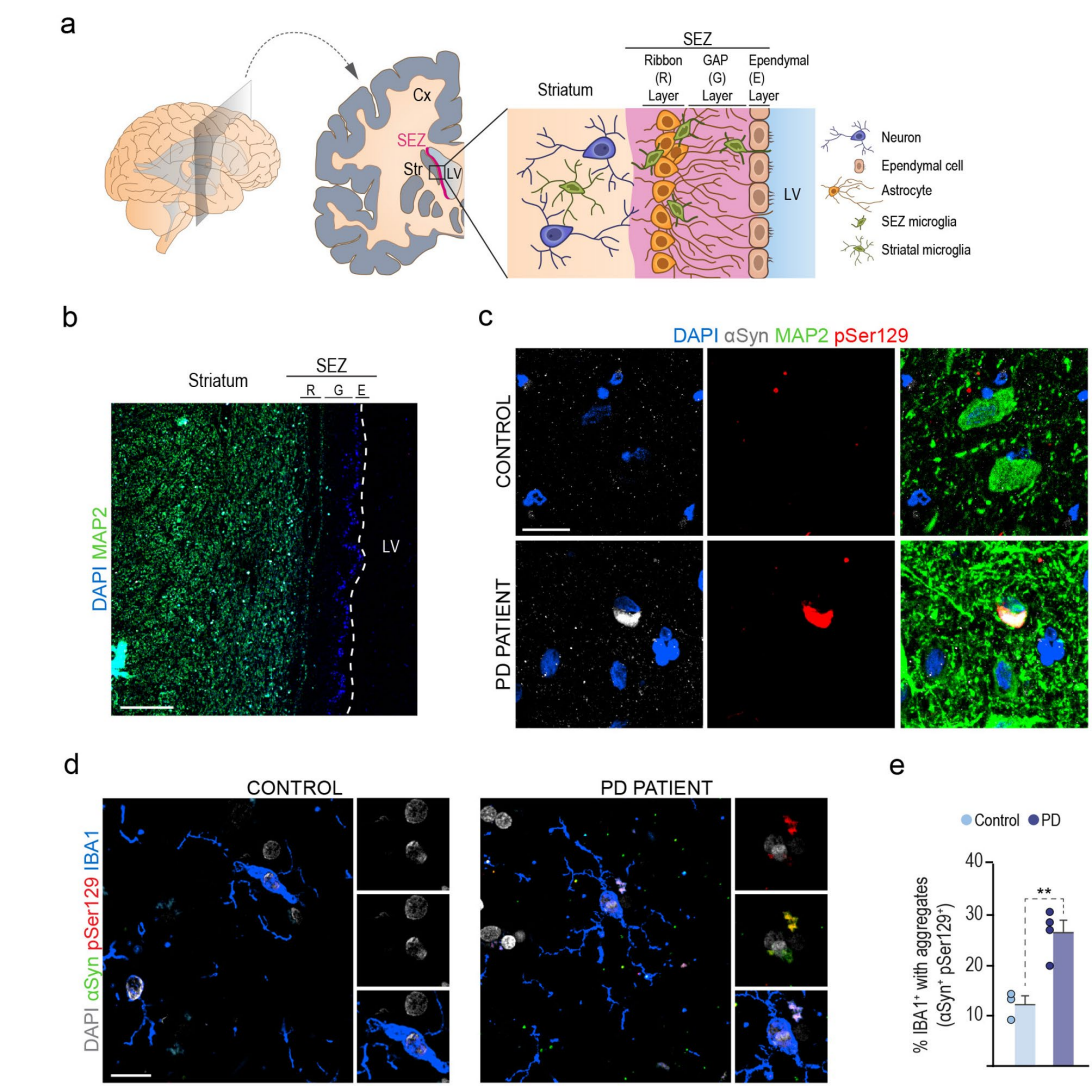
PD patients display human αSyn-pSer129 aggregates in microglial cells. **(a)** Schematic illustration depicting a coronal view of the SEZ in the adult human brain. **(b)** Panoramic view of the human SEZ stained for neuronal MAP2. **(c)** Confocal images showing staining for αSyn (grey), pSer129 (red), and neuronal MAP2 (green) in controls and PD samples. DAPI: blue. **(d)** Confocal images showing staining for αSyn (grey), pSer129 (red), and microglial IBA1 (green) in controls and PD samples. DAPI: grey. **(e)** Quantification of microglia containing αSyn aggregates in controls (*n* = 3) and PD samples (*n* = 4). Data are presented as mean values ± SEM; Student’s *t-test*,* p* < 0.01. Scale bars: b, 240 μm; c, 30 μm and d, 25 μm

Because our data indicated that periventricular microglia can act as a first line of defense at the ependyma-CSF interface, we next decided to explore SEZ microglial cells in *postmortem* human samples by immunostaining PD and non-affected control autopsy brain samples ranging from 65 to 84 years (Fig. 5a, b and Suppl. Table 1) with αSyn and pSer129 αSyn antibodies. We could readily detect pSer129/αSyn-positive aggregates in MAP2-positive neurons in the PD samples (Fig. 5c), but also in periventricular microglia, with similar aggregates that were significantly more numerous in PD samples (Fig. 5d, e). Although these results could be attributed to aggregation of pathological αSyn in microglia, they could also be in line with human periventricular microglia playing an active role in αSyn clearance from the CSF. We decided to explore these possibilities in mice using human LB fractions.

**Fig. 6:**
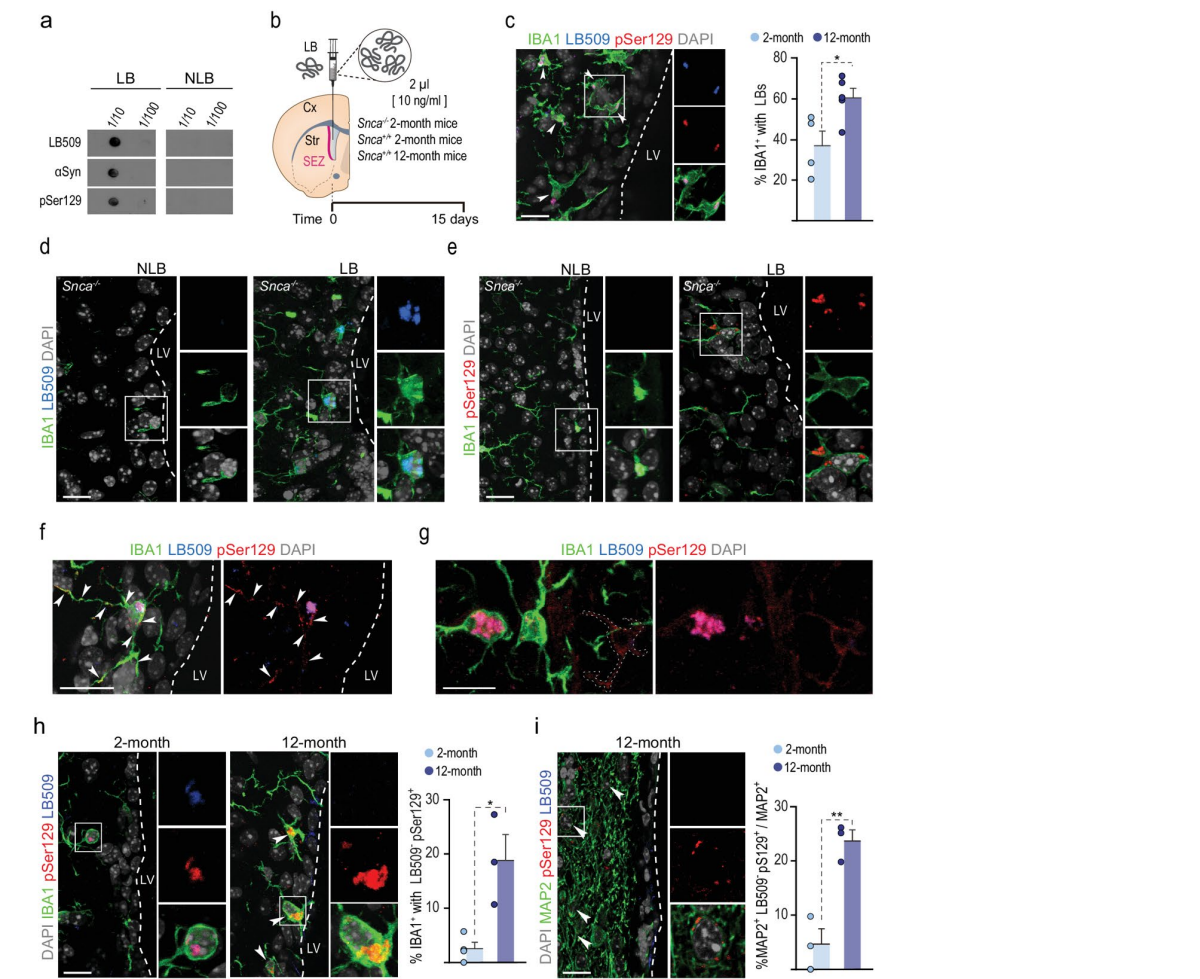
Microglial handling of human LB fractions. **(a)** Dot blot of LB and NLB fractions immunolabeled for αSyn, human αSyn (LB509) and pSer129. **(b)** Schematic representation of the in vivo experiment. **(c)** Left: Confocal images of the SEZ of young mice showing human αSyn (LB509, blue) and pSer129 (red) aggregates in microglia (IBA1, green) in young *Snca*^*−/−*^ mice 15 days after infusion. Right: Quantification of the percentage of microglia containing LBs in 2- (*n* = 4) and 12-month-old (*n* = 5) mice. **d**,** e**. Confocal images of the staining with LB509 (blue) (**d**) and pSer129 (red) (**e**) antibodies in *Snca*^*−/−*^ mice injected with LB and NLB fractions 15 days after infusion, as specificity controls. **f.** Confocal images of the SEZ of aged mice showing human LB509 (blue)/ pSer129 (red) double–positive aggregates and LB509 negative/ pSer129 positive aggregates. **g.** Confocal images of the SEZ of aged mice showing microglia (IBA1, green) displaying LB509 (blue)/pSer129 (red)-positive aggregates near to LB509-negative/pSer129-positive cells. **h.** Confocal images showing microglia (IBA1, green) containing endogenous αSyn aggregates that are LB509-negative/pSer129-positive in 2- and 12-month-old mice injected with LB fractions (left) and quantification of the percentage of microglia with endogenous αSyn aggregates in 2- (*n* = 4) and 12-month-old (*n* = 3) mice. Data are presented as mean values ± SEM; Student’s *t-test*,* p* < 0.05. **i.** Confocal images showing neurons (MAP2, green) containing endogenous αSyn aggregates (LB509-negative/pSer129-positive) in 12-month-old mice injected with LB fractions (left) and quantification of the percentage of neurons with endogenous αSyn aggregates in 2- (*n* = 4) and 12-month-old (*n* = 3) mice. Data are presented as mean values ± SEM; Student’s *t-test*: * *p* < 0.05, ** *p* < 0.01. DAPI: grey. Scale bars: c-f, h and i, 20 μm; g 10 μm

There is an increasing appreciation that PFFs only reproduce specific aspects of LB pathology [58], fostering the usage of aggregated αSyn derived from neurons or αSyn-containing LB fractions. We had previously reported that inoculation of LB-enriched fractions of pathological αSyn purified by sucrose gradient from *postmortem* PD brains into the substantia nigra of young mice initiates a slowly progressive nigrostriatal degeneration that is not detected after inoculation of non-LB (NLB) fractions [9, 10]. We, therefore, decided to explore the short-term effects of the LV infusion of similar LB fractions, containing pathological αSyn, and NLB control fractions isolated from *postmortem* samples (Fig. 6a). We next infused the fractions into the LV of young mice and fifteen days later, we immunostained SEZ-containing sections of the infused mice with antibodies specific to human αSyn (LB509 antibody) and to pSer129-αSyn. We found detectable uptake of the LB-derived human αSyn aggregates by periventricular IBA1-positive microglia (Fig. 6c). In line with our previous observations using PFFs, the percentage of microglia containing LBs was significantly higher in 12- vs. 2-month-old mice (Fig. 6c). To ensure the specificity of the LB509 and pSer129 antibodies employed in the analysis we also performed the infusions in *Snca* null mice. At 15 days after the infusion, labelings for LBs and pSer129 were only found in animals injected with LB, but not NLB fractions, highlighting the specificity of these antibodies (Fig. 6d, e). As with PFFs, we compared the infusions in mice at 2-months and 12-months of age and found LB material within more astrocytes in the latter (Suppl. Figure 7a, b). The data indicated that periventricular microglia can also phagocytize LB-enriched fractions from the CSF and further sustain the idea that, with advancing age, astrocytes react more actively to toxic αSyn, likely due to microglial reduced efficiency.

We next focused on the potential differences in the response of periventricular microglia to PFFs vs. LB fractions. In contrast to PFF infusions, and specifically at 12-months of age, we noticed some microglial cells with LB509^+^/pSer129^+^ aggregates inside that also displayed LB509-negative/pSer129^+^ aggregates, suggesting a potential seeding effect on microglia (Fig. 6f). In addition, we observed pSer129^+^ cells close to LB-phagocytic microglia suggesting a potential spreading effect from microglia at 12-months (Fig. 6g). To delve into this potential prionoid effect, we next analyzed the proportions of SEZ microglial cells and nearby MAP2-positive neurons that were positive for pSer129-αSyn, but negative for LB509 and we observed increased proportions of what appeared to be phosphorylated endogenous αSyn in both cell types in 12-month vs. 2-month-old mice (Fig. 6h, i). Our results suggested a phosphorylation of the endogenous murine αSyn in microglia and in neurons of 12-month-old animals exposed to LB material and a potential spreading among periventricular microglia, in addition to adjacent neurons, at very short times. To assess the potential spread to more distant regions, analyses at durations beyond those examined in this study would be necessary.

**Fig. 7:**
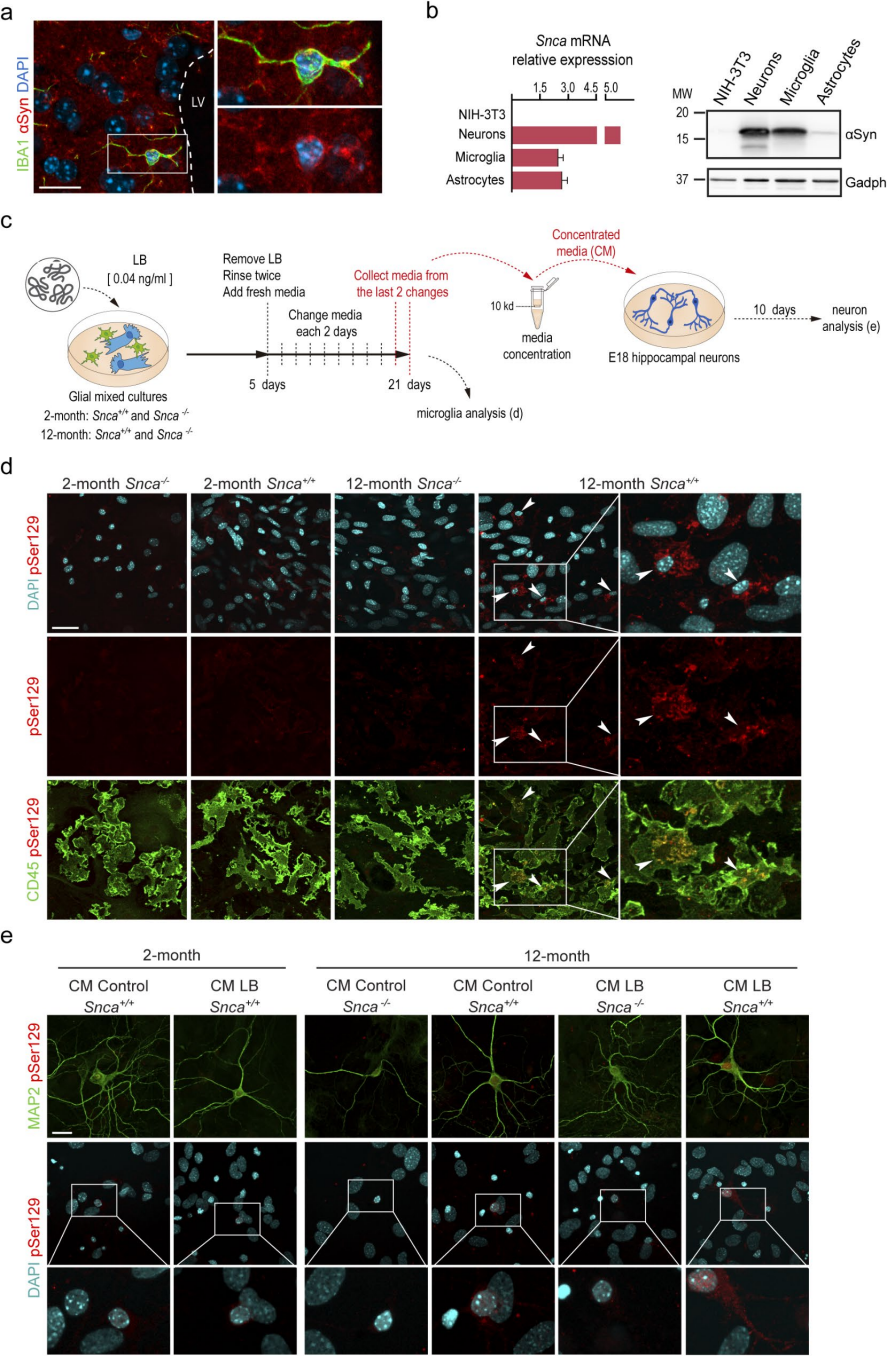
Microglia exert αSyn prion-like effects. **(a)** Confocal images showing αSyn (red) in IBA1-positive microglia (green). DAPI: blue. **(b)** Quantitative RT-PCR analysis showing *Snca* mRNA levels in NIH-3T3 cells, neurons, microglia and astrocytes and representative Western blot showing the relative αSyn protein levels in the same cells. **(c)** Schematic representation of the in vitro experiment to test potential prion-like effects in microglia. **(d)** Representative fluorescence images of endogenous phosphorylated αSyn (pSer129, red) in microglia (CD45, green) in mixed glial cultures obtained from 2- and 12-month-old *Snca*^*+/+*^ and *Snca*^*−/−*^ mice, treated for 5 days with LB-fractions and maintained for 21 days. DAPI: cyan. **(e)** Representative fluorescence images of phosphorylated αSyn (pSer129, red) in hippocampal neuron (MAP2, green) cultures incubated with concentrated conditioned media (CM) obtained from mixed glial cells from 2-month *Snca*^*+/+*^ mice or 12-month-old *Snca*^*+/+*^ and *Snca*^*−/−*^ mice treated with either PBS (control) or LBs. DAPI: cyan. Scale bars: a, 20 μm; d, 40 μm; and e, 30 μm

αSyn prion-like effects have been widely studied in neurons, but similar effects in microglial cells remain elusive. Previous reports have indicated the presence of αSyn in mouse microglial cells [59, 60], and we could readily detect αSyn protein in the intact murine SEZ (Fig. 7a) and other brain regions such as cortex or dentate gyrus (Suppl. Figure 8a, b) using immunofluorescent detection with specific antibodies. To obtain a more direct evidence that LB uptake by microglia could have non-autonomous effects on other cells, we turned to an in vitro system. Expression analysis by RT-PCR and Western blot analysis in dissociates of magnetically isolated brain microglia (CD11b-positive fraction), astrocyte-enriched cultures, and neuronal hippocampal cultures confirmed that microglia have levels of αSyn that are similar to those of neurons and much higher than those of astrocytes (Fig. 7b). We, therefore, established mixed cell cultures from 2- and 12-month-old mice and incubated them for 5 days with LB-fractions or vehicle, then washed the cultures with PBS and allowed them to survive for 21 days, with changes of medium every 2 days, to wash out LB remnants and allow for the internalized LB-fractions to be digested. After 26 days, we could detect pSer129-αSyn in 59.80 ± 4.40% of the microglia in the cultures obtained from 12-, but not 2-month-old animals. We could not detect the signal in cultures derived from 12-month-old *Snca* mutant mice (Fig. 7c, d), indicating that the label corresponded to the phosphorylation of endogenous αSyn. These results suggested a seeding effect of LB αSyn fractions in microglia during aging.

We had also observed potential signs of spreading of the αSyn pathology from periventricular microglia to neurons adjacent to the SEZ (Fig. 6i). Although selective overexpression of *SNCA*^*G420A*^ in microglia reportedly induces nigral neurodegeneration [61], whether deficient αSyn degradation by microglia contributes to the spreading of the pathology remains unknown. We, therefore, decided to analyze whether the uptake of LB fractions by microglia could have a prion-like effect on neurons. To do so, glial mixed cultures obtained from 2- and 12-month-old *Snca*^*+/+*^ and *Snca*^*−/−*^ mice were treated with LB or PBS vehicle for 5 days and washed every 2 days with fresh culture medium for 21 more days. Media were collected from all the cultures in the last two changes, and these conditioned media (CM) were concentrated and added to E18 hippocampal neuron cultures (Fig. 7c). We observed that only the CM from glial cultures of 12-month-old *Snca*^*+/+*^ mice treated with LB-fractions induced the phosphorylation of endogenous neuronal αSyn, as 77.5 ± 2.5% (*n* = 3 different cultures) of the neurons were positive for pSer129-αSyn (Fig. 7e; Suppl. Figure 9a). Collectively, our results indicate that periventricular microglia exert a protective role against aggregated αSyn, clearing it from extracellular spaces and CSF, but that it can, in turn, contribute to the spreading of the αSyn pathology in aged individuals. This latter effect could be observed with LB fractions but not PFFs, highlighting the relevance of αSyn species.

## Discussion

Misfolded αSyn has been proposed to propagate from cell-to-cell in a prion-like fashion, triggering aggregation of αSyn in recipient cells. However, the dissemination of αSyn pathology does not necessarily follow the expected neuroanatomic connectivity suggesting that other mechanisms besides neuron-to-neuron spreading could be involved in the transmission of αSyn aggregates throughout the brain [27]. Here, we have evaluated the potential involvement of periventricular microglia in surveilling the CSF, a recognized natural vehicle fluid for αSyn dissemination. We have taken advantage of the similar molecular profiles of DAM and PAM and the presence of the latter in the SEZ neurogenic niche [35] to test in vivo how this non-homeostatic microglia senses and handles normal and aggregated toxic αSyn under physiological conditions. Furthermore, the location of periventricular microglia has allowed us to evaluate short-term responses to αSyn in vivo to reveal that microglia handle and clear aggregated αSyn present in the CSF very rapidly in the absence of damage. We also show that this clearance is less efficient in aged mice and that both aged mice and pathological human microglia accumulate phosphorylated αSyn. Exposure of microglia in aged mice to LB-fractions containing αSyn, but not to in vitro generated αSyn PFFs, results in spreading to other microglial cells and to neurons at very short times. Our data indicate that periventricular microglia act as a first line of defense for the handling of αSyn present in the CSF and that when their capacities wear off with ageing, they can contribute to the spreading of the synucleinopathy.

Clearance and degradation of aggregated αSyn are key to prevent neuronal uptake and cell-to-cell transfer. The aging process in microglia reportedly results in alterations in their surveillance phenotype, with reduced motility and impaired phagocytic and lysosome-degradative capabilities [52, 62]. Here, we demonstrate that young microglia can uptake, phosphorylate and degrade misfolded αSyn through the autophagy-lysosomal pathway. The impairment in αSyn clearance capability in microglia with time leads to the presence of long-lasting aggregates in their cytoplasm and the extracellular propagation of pathological αSyn to other cell types, such as neurons. Interestingly, some genetic risk factors for PD are related to lysosomal activity. Genome-wide association studies (GWAS) show that gene sequence variations related to degradation pathways such as *LRRK2*, *GBA*, *CHMP2B*,* TMEM175*,* SCARB3*,* or BAG3* are associated with PD [63]. Mutations in the *LRRK2* gene are the most common in both familial and sporadic PD, and LRRK2 plays a role in autophagosome/lysosome degradation pathways [64–66]. Another major risk factor for PD is mutations in *GBA* [67], which encodes for glucocerebrosidase, an enzyme involved in lipid hydrolysis. Mutations in this gene cause impaired lysosomal function and αSyn accumulation and aggregation [68, 69]. Most of the published studies on the role of gene mutations in PD pathogenesis focus on neurons. Thus, it would be interesting to investigate how the different mutations alter the degradation capacity of microglia and PD progression. Inhibition of glucocerebrosidase in microglia is sufficient to impair their physiological ability to protect neurons against oxidative stress and neurotoxic stimuli [70].

In this work, we have used in vitro generated PFFs as a model of misfolded αSyn to investigate the role of microglia in the clearance of aggregated αSyn. In parallel, we have also used aggregated αSyn obtained from *bona fide* LBs extracted from PD brain tissue to model naturally occurring inclusions. Using LB extracts, we could recapitulate our observations with PFFs. In addition, we observed a prion-like effect of αSyn in microglia not observed with PFFs. The striking differences obtained between in vitro and in vivo generated αSyn seeds highlight their differential pathogenicity. αSyn PFFs generated in vitro have different conformations compared to αSyn extracted from LB brains [71–73]. Pathologic αSyn aggregates obtained from LBs harbor extensive posttranslational modifications, including truncation, phosphorylation, ubiquitination, nitration and sumoylation that affect αSyn aggregation and spreading [74, 75]. Thus, we propose that αSyn obtained from LBs may represent a more reliable source to analyze the initiation and progression of PD at first stages.

In our study, we describe disturbed αSyn clearance in microglia in elderly mice. Aging also induces a shift in the homeostatic state of microglia towards an active/reactive one. This process starts at early stages in aging, as 12-month-old mice microglia present functional impairments, suggesting a temporal progression likely to worsen with further increasing age. In an active state, microglia release pro-inflammatory cytokines and produce reactive oxygen species (ROS) such as NO and superoxide [76], helping create a detrimental microenvironment for neurons [61]. CD22 is a sialic acid-binding immunoglobulin-like lectin, is a negative phagocytosis regulator upregulated on aged microglia. Its blockade promotes the clearance of αSyn fibrils in vivo, among other substrates, but also partially reverses the transcriptional signature of aged microglia towards that of a more homeostatic state [77]. Interestingly, CD22 is upregulated upon αSyn uptake by microglia, exacerbating its phagocytic exhaustion and its inflammatory state. Thus, rejuvenating microglia by blocking CD22 in combination with anti-inflammatory drugs may be a potential therapeutic strategy to target microglia and prevent cell-to-cell transfer, neurodegeneration, and PD progression.

In summary, using a new model to study αSyn uptake and spreading in vivo by delivering aggregated αSyn in the LV, we demonstrate that periventricular microglia efficiently clear aggregated αSyn from the CSF. However, αSyn uptake and degradation are severely impaired with age. We suggest that microglia serve as the initial barrier against aggregated αSyn. However, when microglial capacity is exceeded, its ability to provide neuroprotection diminishes, thereby facilitating the transfer and propagation of αSyn between cells. Therefore, targeting microglia by enhancing their phagocytic/degradative function while limiting inflammation could represent a promising therapeutic approach for PD.

## Experimental procedures

### ***Animals***

Generation of *Snca* mutant mice and transgenic mice containing human wild-type αSyn cDNA under the control of TH promoter (TH-hαSyn) and their genotyping by PCR have been described elsewhere [38, 78]. *Cx3cr1-eGFP* knock-in mice (*B6.129P2(Cg)-Cx3cr1*^*tm1Litt*^*/J*) were obtained from the Jackson Labs. *Snca* mutant mice and their controls are on a mixed C57BL6/Sv129 background. All animals were gender group-housed with standard pellet food and tap water *ad libitum*. Either male or female mice were indistinctly used in the experiments. Animal handling and all experimental procedures were carried out in accordance with European Union 2010/63/UE and Spanish RD53/2013 directives, following protocols approved by the ethics committee on experimental research of the Universidad de Valencia.

### ***Human tissue and analysis***

Four autopsies of patients diagnosed with PD and three patients considered as controls were analyzed; controls were patients with no history of neurological disease or with a cause of death unrelated to any neurological condition. Patients were diagnosed following the *Movement Disorder Society criteria for Parkinson’s disease,* which establish three levels of diagnostic confidence: Definite, Probable, and Possible. The diagnoses of Possible and Probable PD rely solely on clinical criteria, while the diagnosis of Definite PD requires neuropathological confirmation. All samples included in this study were Definite PD. Sample processing was carried out with prior informed consent signed by their relatives and authorization from the ethics committee of the Hospital Clínico San Carlos (Ref. 16/398-E, Ref Biobank 22001). Samples were age-matched, ranging from 65 to 84 years of age, and the presence of co-pathologies is detailed in **Supp. Table 1**. Briefly, autopsies were performed 4–8 h after death following Hospital San Carlos’s standard protocol and in compliance with Spanish regulations. Tissue samples were fixed in 10% buffered formalin. Each hemisphere was sectioned in 1 cm thickness coronal sections and pieces containing the LV and the SEZ selected and prepared for paraffin embedding. Tissue was sectioned into 6 μm slices using a microtome (Leica). Epitopes were unmasked in 10 mM sodium citrate buffer, pH 6, at 96 °C for 30 min, and autofluorescence was quenched with TrueBlack^®^. Then, all samples were blocked in 10% FBS, 0.2% Triton X-100 in PBS for 1 h and incubated with rabbit antibodies to pS129-αSyn (1:200, Abcam, Ab51253), mouse antibodies to αSyn (1:250, Abcam, ab 610786), goat antibodies to IBA1 (1:500, Abcam, ab5076) and chicken antibodies to GFAP (1:500, Millipore), MAP2 (1:1000, Abcam, ab5392) or GFAP (1:1000, Thermo Fisher-Invitrogen, PA1-10004), alone or in different combinations for 72 h at 4 °C. After several washes, sections were incubated in the appropriate Alexa-Fluor secondary antibody for 24 h at 4 °C and counterstained with DAPI. Sections were mounted in Fluorsave and observed in an Olympus AF2000 confocal microscope. The area analyzed, containing the SEZ, included 200 μm from the LV.

### ***Production of PFFs***

PFFs were made following the Michael J. Fox Foundation protocols. Endoclear mouse recombinant αSyn (AS-56082-500; AnaSpec) was dissolved in sterile 50 mM Tris-HCl, 150 mM NaCl, pH 7.5 at a concentration of 5 mg/ml and filtered through 100 kDa MW-cut-off Amicon filters (MRCF0R100; Millipore). Concentrations were determined by measuring absorbance at 280 nm using a NanoDrop. αSyn fibrils were formed by incubating 3 mg/ml filtered monomeric solution in an orbital shaker (Eppendorf Thermomixer) at 37 ºC for 7 days at 1,000 rpm. Labeling of αSyn fibrils and monomers with Alexa Fluor 555 was performed with microscale labeling kits (A3007; Molecular Probes) according to the manufacturer’s instructions. To eliminate the unreacted dye, fibrils were centrifuged twice at 20,000*g* for 30 min and the pellet was resuspended in PBS. Labeled fibrils at a concentration of 1 mg/ml were sonicated with a Bioruptor, a high-powered water bath sonicator, at high power for 20 cycles (30s ON, 30s OFF). To avoid repeated freeze and thaw cycles, resulting PFFs were aliquoted and stored at -80 ºC. Additional quality control experiments to validate the successful conversion of αSyn monomers to fibrils and PFFs were performed, including sedimentation assays (to confirm fibrillation) and TEM analyses (to visualize the size and morphology of aggregates). Briefly, in the sedimentation assay, 10 µg polymerized fibrils were centrifuged at 100,000*g* for 30 min at 25 ºC. The supernatant, containing unreacted monomers, and the pellet, composed of aggregated fibrils, were resolved by SDS-PAGE. For TEM analysis, fibrils and PFFs were deposited onto 300 mesh carbon coated copper grids for 10 min. The grids were quickly transferred through three drops of Tris-HCl (50 mM pH 7.4) rinse, then negatively stained with 2% phosphotungstic acid pH 7.0 for 3 min. Finally, the excess of staining was blotted off with Whatman filter paper. Grids were examined in a JEM 1010 electron microscope (Jeol). The final size of the PFFs was determined using the linear (point to point) utility in the AMT Image Capture Engine software provided with the camera.

### ***Purification of LBs from human PD brain***

LB purification was conducted as previously described [8–11]. Briefly, the substantia nigra was dissected from frozen *postmortem* midbrain samples of 3 patients with sporadic PD exhibiting conspicuous nigral LB pathology on neuropathological examination. Tissue was homogenized in 9 vol (w/v) ice-cold MSE buffer (10 mM MOPS/KOH, pH 7.4, 1 M sucrose, 1 mM EGTA, and 1 mM EDTA) with protease inhibitor cocktail (Complete Mini; Boehringer Mannheim) with a motor-driven homogenizer. For LB purification, a sucrose step gradient was prepared by overlaying 2.2 M with 1.4 M and finally with 1.2 M sucrose in volume ratios of 3.5:8:8 (v/v). The homogenate was layered onto the gradient and centrifuged at 160,000*g* for 3 h using a SW32.1 rotor (Beckman Coulter). Twenty fractions of 500 µl were collected from each gradient from the top (fraction 1) to the bottom (fraction 20) and analyzed for the presence of αSyn aggregates by filter retardation assay. Fractions containing insoluble aggregates were considered as LB fractions, and remote non-containing insoluble aggregates as non-LB fractions (NLB). These fractions were further characterized by immunofluorescence, αSyn ELISA quantification, and EM. Before use, NLB and LB fractions were validated for αSyn and pSer129 by dot blot. In all cases, samples were bath-sonicated for 5 min before applications.

### ***CSF collection and human αSyn quantification***

CSF was collected from the cisterna magna by a puncture technique. In brief, 2-month-old mice were deeply anesthetized with ketamine (100 mg/kg) and xylazine (10 mg/kg). Mice were placed prone, and their cisterna magna was surgically exposed. The meninges were penetrated with a sharp glass capillary and CSF was obtained. To avoid blood contamination, surgery was conducted carefully, and CSF samples were centrifuged at maximum speed for 15 min. The presence of αSyn was assessed by immunoblotting, and the concentration of hαSyn was determined using a specific ELISA kit (KHB0061; Invitrogen), according to the manufacturer’s instructions.

### ***Stereotaxic intra-cerebroventricular infusions***

Unilateral infusion into the LVs was conducted under isoflurane anesthesia in a stereotaxic frame. 2 µl of PFFs (0.1 mg/ml), αSyn monomers (0.1 mg/ml), LB fractions (10 ng/ml) or NLB fractions, were delivered into the ventricle (-0.1 mm AP, 0.8 mm ML, -2.6 mm DV) at a constant flow rate of 0.5 µl/min with a Hamilton syringe in 2- and 12-month-old mice. After delivery, the needle was kept in the same position for 5 min to avoid CSF reflux. At 2 days or 15 days after surgery, mice were transcardially perfused, and the brains collected and processed for histochemical examination. To analyze the phagocytic capacity of microglial cells, 2 µl of 1.0 μm red fluorescent microspheres (FluoSpheres, F-8821; Molecular Probes) were stereotactically infused into the LV following the protocol described above. Three days after infusion, animals were euthanized and the brains were collected. The ipsilateral side of the infusion was used for flow cytometry analyses, and the contralateral side was immersion-fixed for immunohistochemical analyses in whole-mount-en-face preparations.

### ***Immunohistochemistry***

Mice were deeply anesthetized and transcardially perfused with 4% paraformaldehyde (PFA) in 0.1 M phosphate buffer pH 7.4 (PB), and brains were processed for vibratome sectioning at 40 μm or dissected to obtain whole-mount-en-face preparations of the SEZ as described previously (Belenguer et al., 2016). Sections were blocked in PB containing 10% FBS and 0.2% Triton X-100. Sections were then incubated with rabbit antibodies to pS129-αSyn (1:400, Abcam, ab59264 and Abcam, ab51253), P2RY12 (1:500, AnaSpec, AS55043A), CD68 (1:500, Abcam, ab125212) or β-catenin (1:100, Cell Signalling Technology, 9587), mouse antibodies to αSyn (1:500, BD, 610786), human αSyn (LB509, 1:500, Abcam, ab27766), S100β (1:500, Sigma-Aldrich, S2532), acetylated tubulin (1:500, Sigma, T6793) or γ-tubulin (1:200, Sigma, T6557), goat antibodies to IBA1 (1:500, Abcam, ab5076), chicken antibodies to GFAP (1:800, Millipore, AB5541) or MAP2 (1:500, Abcam, ab5392) and rat antibodies to dectin 1 (CLEC7A) (1:50, InvivoGen, 6114-43-01), alone or in different combinations for 24–48 h at 4 °C. After several washes, the sections were incubated for 1 h at room temperature with appropriate fluorescently-labeled secondary antibodies: Alexa Fluor^®^ 488 Donkey Anti-mouse, Alexa Fluor^®^ 488 Donkey Anti-rabbit, Alexa Fluor^®^ 647 Donkey Anti-mouse, Alexa Fluor^®^ 647 Donkey Anti-rabbit, Alexa Fluor^®^ 647 Donkey Anti-goat (1:800 Molecular Probes), Alexa Fluor^®^ 488 Donkey Anti-chicken, Cy3 Donkey Anti-rabbit, Cy3 Donkey Anti-goat (1:800 Jackson ImmunoResearch Laboratories). DAPI (1 µg/ml, 4 min, Sigma-Aldrich) was used for counterstaining. Images were acquired and processed using an Olympus Fluoview FV10i confocal microscope and the FV10-ASW 2.1 viewer software.

Confocal images at 100x magnification were collected every 1 μm covering all the open LV ventricular wall. Every region of interest in microscope images was 1,250 μm length x 50 μm width x 15 μm thick (on average). The SEZ was fully included in 50 μm from the LV and only cell nuclei included in this distance were considered in quantifications. Immunostained sections (at least 5 slices *per* experiment *per* mouse) were photographed using an Olympus FV-10i confocal microscope. Separate images were taken with restrictive excitation filters in each fluorescent channel every 1 μm. Subsequently, individual cells were identified across the tissue and quantitative analyses were performed on high-resolution image stacks. At least 10 sections were analyzed in every brain slice. In this way, the entire ventricular surface was scrutinized in detail. ​​Quantification of microglial activation was performed by measuring the number of individual IBA1^+^ microglial cells and the area occupied by all of them with ImageJ [79] (version 2.1.0/1.53c) software using a single automated macro-script to quantitate areas. The IBA1^+^ area was normalized to the total tissue area using the DAPI signal.

### ***Electron microscopy***

Mice were transcardially perfused with saline followed by 4% PFA and 0.1% glutaraldehyde (EM grade, Electron Microscope Science) in PB. The brain was removed, post-fixed overnight in 4% PFA, and coronally sectioned with a vibratome at 50 μm. Sections were pre-incubated in blocking solution (10% FBS and 0.1% Triton X-100 in PB 0.1 M) and then incubated in primary antibody goat anti-IBA1 (1:500, Wako, 019-19741) prepared in blocking solution, on an orbital shaker for 24 h at 4 ºC. After several washes in PB, tissue sections were incubated in biotinylated rabbit anti-goat IgGs (1:1,000, Vector Laboratories, BA5000) for 1 h at RT. After washing, sections were incubated with an avidin-biotin-peroxidase complex (ABC, Elite Vector Laboratories), washed and revealed with 0.05% diaminobenzidine (DAB) and 0.01% hydrogen peroxide (Sigma) in PB. The DAB-stained sections were further processed for electron microscopy. Briefly, tissue was osmicated (1% OsO_4_ in PB, 20 min), dehydrated in graded alcohols to propylene oxide, and plastic-embedded flat in Durcupan (Sigma). To study selected microglial cells in contact with the ventricle, serial 1.5-μm sections were cut with a diamond knife and stained with 1% toluidine blue. Subsequently, the area of interest was trimmed, and ultrathin Sects. (50–70 nm) were obtained from this material, stained with lead citrate, and examined in a JEM 1010 (Jeol) electron microscope.

### ***Primary cell cultures and treatments***

For mixed cell cultures (astrocytes and microglia) and pure microglia cultures, cortices, or SEZs from 2- and 12-month-old C57/Bl6 mice were dissected, minced, and enzymatically digested using the Neural Tissue Dissociation kit (P) (130-092-628, Miltenyi Biotec) in a gentleMACS Octo Dissociator with heaters (Miltenyi Biotec; 37 ºC_ABDK_01 program). Digestion was diluted with 3 ml of washing solution (0.6% glucose, 0.1% NaHCO_3_, 5 mM HEPES, 2 mM L-glutamine, 0.4% BSA, 1X antibiotic/antimicotic in DMEM/F-12) (all from Invitrogen) and digested pieces were mechanically dissociated by pipetting up and down 20 times through a plastic Pasteur pipette. Cell suspension was filtered through a 40 μm nylon filter and then centrifuged. To prepare primary mixed glial cell cultures, the cell pellet was resuspended in complete medium (DMEM supplemented with 10% heat-inactivated FBS, 2 mM L-glutamine, 1 mM sodium pyruvate, 100 U/ml penicillin, and 100 mg/ml streptomycin) and seeded on poly-D-lysine coated coverslips. Alternatively, the single-cell suspension was subjected to immunomagnetic CD11b positive selection using CD11b microbeads (130-049-601, Miltenyi Biotech) in combination with an OctoMACS following manufacturer´s instructions to obtain a microglia-rich fraction (CD11b^+^ fraction) and an astrocyte rich fraction (CD11b^−^ fraction). Purified CD11b^+^ microglial cells were seeded in poly-D-lysine (100 µg/ml)-treated plates and cultured in TIC medium (DMEM/F12 containing 100 units/ml penicillin/streptomycin, 2 mM L-glutamine, 5 µg/ml N-acetyl cysteine, 5 µg/ml insulin, 100 µg/ml apo-transferrin, 100 ng/ml sodium selenite, 2 ng/ml TGF-β2, 100 ng/ml murine IL-34, 1 µg/ml heparan sulfate). Cells were maintained at 37 ºC and 5% CO_2,_ changing the media every 2 days. For primary neuronal cultures, hippocampal neurons were prepared from E18 wild-type and *Snca* null mice. Hippocampi were isolated stereoscopically and enzymatically digested with 12 U/ml papain solution (Worthington) for 15 min at 37 ºC. Dissociated hippocampal neurons were plated on coverslips or tissue culture plates coated with poly-D-lysine (Sigma) at a 50,000 cells/cm^2^ density and cultured in Neurobasal medium containing B27 supplement, L-glutamine and penicillin/streptomycin (Life technologies). Neurons were maintained by changing half of the media every 2 days.

### ***Confirmation of PFFs seeding capacity and toxicity***

Neurons cultured for 5 days were treated with 1 µg/ml PFFs (or PBS as control) for only 24 h or for 10 to 14 days. At the end of the treatment, neurons were scraped and collected in 1% Triton X-100 in Tris-buffered saline (TBS) (50 mM Tris HCl, 150 mM NaCl, pH 7.4) and protease and phosphatase inhibitor cocktail at 4 °C. Lysates were sonicated and centrifuged at 100,000*g* for 30 min. The supernatant (soluble fraction) and the pellet (insoluble fraction) were collected. The pellet was washed and resuspended in 2% SDS in TBS. Both fractions were analyzed by immunoblotting. On the other hand, neurons were fixed with 2% PFA for 20 min at RT, blocked in 10% FBS in 0.1% Triton X-100 PBS for 1 h at RT and then incubated with rabbit anti-MAP2 (1:800, Santa Cruz, SC-20172), mouse anti-NeuN (1:400, Millipore, MAB377) or mouse anti-pSer129 (1:500, BioLegend, 825702) overnight. After several washes, cells were incubated for 1 h at RT with the appropriate secondary antibodies (1:800, Molecular Probes). DAPI (1 µg/ml, 5 min, Sigma) was used for counterstaining. The number of neurons per field was estimated in 30 different fields.

### ***Immunoblotting***

For Western blotting, cells were lysed in ice-cold RIPA buffer (50 mM Tris HCl, 150 mM NaCl, 1 mM MgCl_2_, 1.0% (v/v) NP-40, 0.5% (w/v) sodium deoxycholate, 1 mM EDTA, 0.1% (w/v) SDS, pH 7.4) supplemented with phosphatase and protease inhibitors and total protein concentration was determined using a BCA kit (BCA Protein Assay-Kit, ThermoScientific, Sweden). Proteins were resolved by SDS-PAGE and transferred to nitrocellulose membranes (Bio-Rad) using the TransBlot Turbo system from Bio-Rad. For dot blot, 5 µl of serial diluted LB and NLB fractions were spotted directly onto a nitrocellulose membrane. Membranes were blocked for 1 h with skim milk at 3% (w/v) in TBS-T, followed by incubation with mouse primary antibodies to human-αSyn LB509 (1:500, Abcam, ab27766), αSyn (1:500, BD, 610786), pS129 αSyn (1:500, Abcam, ab59264), α-tubulin (1:100, Sigma, T9026) and GAPDH (1:500, Millipore, mAb 374) or rabbit primary antibody to pS129 αSyn (1:500, Abcam, ab59264) overnight. Then, membranes were washed, incubated with appropriated secondary peroxidase-conjugated antibodies (1:1,000 of goat anti-mouse-HRP or mouse anti-rabbit-HRP from Dako and Santa Cruz, respectively) for 1 h and reacted by chemi-luminiscence (SuperSignal, Thermo Fisher Scientific).

### ***Flow cytometry analyses***

For in vivo phagocytic assay with red fluorescent microspheres, SEZ single cell suspensions obtained from young and aged mice 3 days after red fluorescent microsphere infusion were pelleted (300*g*, 10 min), resuspended in 100 µl blocking buffer (HBSS without calcium and magnesium, 10 mM HEPES, 2 mM EDTA, 0.1% glucose, 0.5% BSA) and incubated with primary antibodies CD45-BUV395 (1:200, BD, 565967), CD11b-APC (1:100, BD, 553312), DAPI 50 µg/ml (1:500, Sigma, D9542) for 30 min at 4 ºC. After washing with 1 ml blocking buffer, labeled samples were centrifuged (300*g*, 10 min, at 4 ºC) and resuspended in 0.5 ml blocking buffer. Cells were analyzed using a LSR-Fortessa (BD) with 355, 561 and 640 nm lasers. For e*x vivo* phagocytosis assays with PFFs, brain single-cell suspensions from young and aged mice were pelleted (300*g*, 10 min), resuspended in 100 µl complete medium containing 1 µg/ml PFFs and incubated at 37 ºC for 5 h. Cells were washed with blocking buffer to remove residual PFFs and then incubated with antibodies as before. After washing with 1 ml of blocking buffer, labelled samples were centrifuged (300*g*, 10 min, at 4 ºC) and resuspended in 0.5 ml blocking buffer. Cells were analyzed using a LSR-Fortessa (BD) with 355, 561, and 640 nm lasers. The phagocytic index was calculated by dividing the median fluorescence intensity (MFI) of each sample by the MFI of the fluorescence minus one (FMO) of its respective group (young or aged). Afterwards, a fold change analysis was performed to determine the magnitude of the observed alteration.

### ***In vitro PFFs uptake assay***

To analyze PFF uptake by microglia and astrocytes, confluent mixed cell cultures or pure microglia cells grown for 7 days were treated with either monomeric αSyn or PFFs at a concentration of 1 µg/ml for 24 h and then fixed with 2% PFA. Cells were blocked in 10% FBS and 0.01% Triton X-100 PB and then incubated with chicken anti-GFAP (1:800, Millipore, ab55414), rat anti-CD45 (1:400, BD, 5530076) and mouse anti-pSer129 (1:500, BioLegend, 825702) antibodies. After several washes, cells were incubated for 1 h at RT with Alexa 488-donkey anti-rat, Alexa 488-donkey anti-mouse, Alexa 647-donkey and anti-chicken (1:800, Molecular Probes) secondary antibodies. Finally, cells were counterstained with DAPI. PFF endocytosis in living cells was imaged by incubating pure microglial cell cultures with 40 µM LysoTracker Deep Red (L12492, Invitrogen) for 30 min, followed by 1 µg/ml PFFs and immediately visualized under an Olympus Fv10i confocal inverted microscope. Serial images were obtained every 5 min for 30 min. Alternatively, cells were treated with PFFs for 24 h, preincubated with LysoTracker Deep Red, and then analyzed. Colocalization with LysoTracker was quantified using JACoP and Manders overlap coefficient.

### ***In vitro degradation assay***

Primary mixed glial cultures were treated with 1 µg/ml PFFs for 3 h at 37 ⁰C and then rinsed twice with PBS. A set of samples was fixed with 2% PFA (initial PFF uptake) for 20 min at RT, while another set of samples was maintained for 8 days to allow PFF degradation and subsequently fixed. Finally, cells were immunostained for the microglial cell marker CD45 as mentioned before. Images were acquired under an Olympus Fv10i confocal inverted microscope. 3D image stacks were preprocessed with a Fiji [80] macro to generate 2D images through maximum projection. Then, CD45-positive cells were then annotated in QuPath (version 0.5.1) [81] for PFF quantification, and the PFF mask associated with each microglial cell was determined using the QuPath-integrated ImageJ’s Moments thresholding algorithm [79]. The final readout for each cell was the PFF area, measured in squared pixels. At least 30 cells *per* condition were analyzed.

### ***Prion-like effect in microglial cells***

Confluent primary mixed glial cultures were treated with LBs at 0.04 ng/ml or vehicle for 5 days, rinsed twice with PBS and then, fresh media was added and replaced every 2 days for 21 days. Media from the last two changes was collected and concentrated 22.5x using 10 kDa MW-cut-off Microcon filters (MRCPRT010; Millipore) to test spreading in hippocampal neuron cultures. To analyze seeding in microglia, cultures were fixed after 21 days and stained with antibodies to pSer129 αSyn (BioLegend) and CD45 (BD), as mentioned above. To analyze spreading from microglia, concentrated media obtained above was diluted 1:40 in neuronal medium and added to hippocampal neuron cultures for seeding capacity. After 10 days, hippocampal neuron cultures were fixed and immunostained for pSer129 αSyn (BioLegend) and MAP2, as described previously.

### ***Gene expression analysis***

Immunomagnetically purified CD11b^+^ microglial cells from young and aged mice were lysed with RLT plus buffer and the DNA extracted with a RNeasy Plus Mini Kit (QIAGEN) following the instructions of the manufacturer. The RNA obtained was quantified using the Qubit^®^ RNA HS Assay Kit (Thermo Fisher) in a Qubit Fluorimeter (Thermo Fisher). For quantitative RT-PCR experiments, a total amount of 50–100 ng of RNA was retro-transcribed to cDNA using the PrimeScriptTM RT-PCR Kit (Clontech) according to the manufacturer instructions. Snca expression analysis was assessed using 5–10 ng of cDNA, specific Taqman probes (Applied Biosystems) and the Premix Ex Taq™ (Probe qPCR) Kit (Clontech). RT-qPCR was performed in a Step One Plus PCR device (Applied Biosystems). The expression level was obtained by relative quantification (2(–ΔΔCt)) using constitutive expression of Gapdh and 18S genes as housekeeping endogenous controls.

### ***Statistical analyses***

All statistical tests were performed using GraphPad Prism Software, version 5.00 for Windows. Analyses of significant differences between means were assessed using the unpaired or paired two-tailed Student’s *t-test* or one-way ANOVA with Tukey post-hoc test when appropriate. When comparisons were carried out with relative values (normalized values and percentages), data were first normalized by using a log or arcsin transformation, respectively. All p-values lower than 0.05 were considered statistically different and referred to as *p < 0.05, **p < 0.01, and ***p < 0.001. Data are always presented as the mean ± standard error of the mean (SEM). The number of experiments carried out with independent cultures/animals (n) is either shown as dots in the graphs or listed in Figure Legends.

## Electronic supplementary material

Below is the link to the electronic supplementary material.

Supplementary Material 1

Supplementary Material 2

## Data Availability

The authors declare that the data supporting the findings of this study are available within the paper and its supplementary information files.
