# Supplementary material for: Age-dependent progression from clearance to vulnerability in the early response of periventricular microglia to α-synuclein toxic species
Source: Mol Neurodegener. 2025 Mar 5;20:26. doi: 10.1186/s13024-025-00816-1 (PMC11881471; doi:10.1186/s13024-025-00816-1)
Supplement: Supplementary file 1 — Supplementary Material 1 [file 13024_2025_816_MOESM1_ESM.pdf]

Age-dependent progression from clearance to vulnerability in the response of periventricular glia to α-synuclein toxic species

Supplementary information

Mª Salomé Sirerol-Piquer[1-3*#], Ana Perez-Villalba[1-3*$], Pere Duart-Abadia[1-3*], Germán Belenguer[1-3], Ulises Gómez-Pinedo[4], Laura Blasco-Chamarro[1-3], Pau Carrillo-Barberà[1-3], Azucena Pérez-Cañamás[2,3], Victoria Navarro-Garrido[5-6], Benjamin Dehay[7], Miquel Vila[1,8-9], Javier Vitorica[1,5-6], Francisco Pérez-Sánchez[1-3], and Isabel Fariñas[1-3#]

[1]Centro de Investigación Biomédica en Red de Enfermedades Neurodegenerativas (CIBERNED), Spain.
[2]Departamento de Biología Celular, Biología Funcional y Antropología Física and [3]Instituto de Biotecnología y Biomedicina (BioTecMed), Universidad de Valencia, Burjassot, Spain.
[4]Laboratory of Neurobiology, Institute of Neurosciences, Hospital Clínico San Carlos Health Research Institute, Universidad Complutense de Madrid, Madrid, Spain.
[5]Instituto de Biomedicina de Sevilla (IBiS) and [6]Departamento Bioquímica y Biología Molecular, Universidad de Sevilla, Seville, Spain.
[7]Univ. Bordeaux, CNRS, IMN, UMR 5293, F-33000 Bordeaux, France
[8]Neurodegenerative Diseases Research Group, Vall d´Hebron Research Institute, Autonomous University of Barcelona, Barcelona, Spain
[9]Catalan Institution for Research and Advanced Studies (ICREA), Barcelona, Spain.

[$]Present address: Departamento de Neuropsicobiología, Facultad de Psicología. UCV, Valencia, Spain.

*These authors contributed equally

Addresses for correspondence: Isabel Fariñas and Mª Salomé Sirerol-Piquer.
Phones: +34-9635-43784 and 44175.
E-mails: isabel.farinas@uv.es and M.Salome.Piquer@uv.es

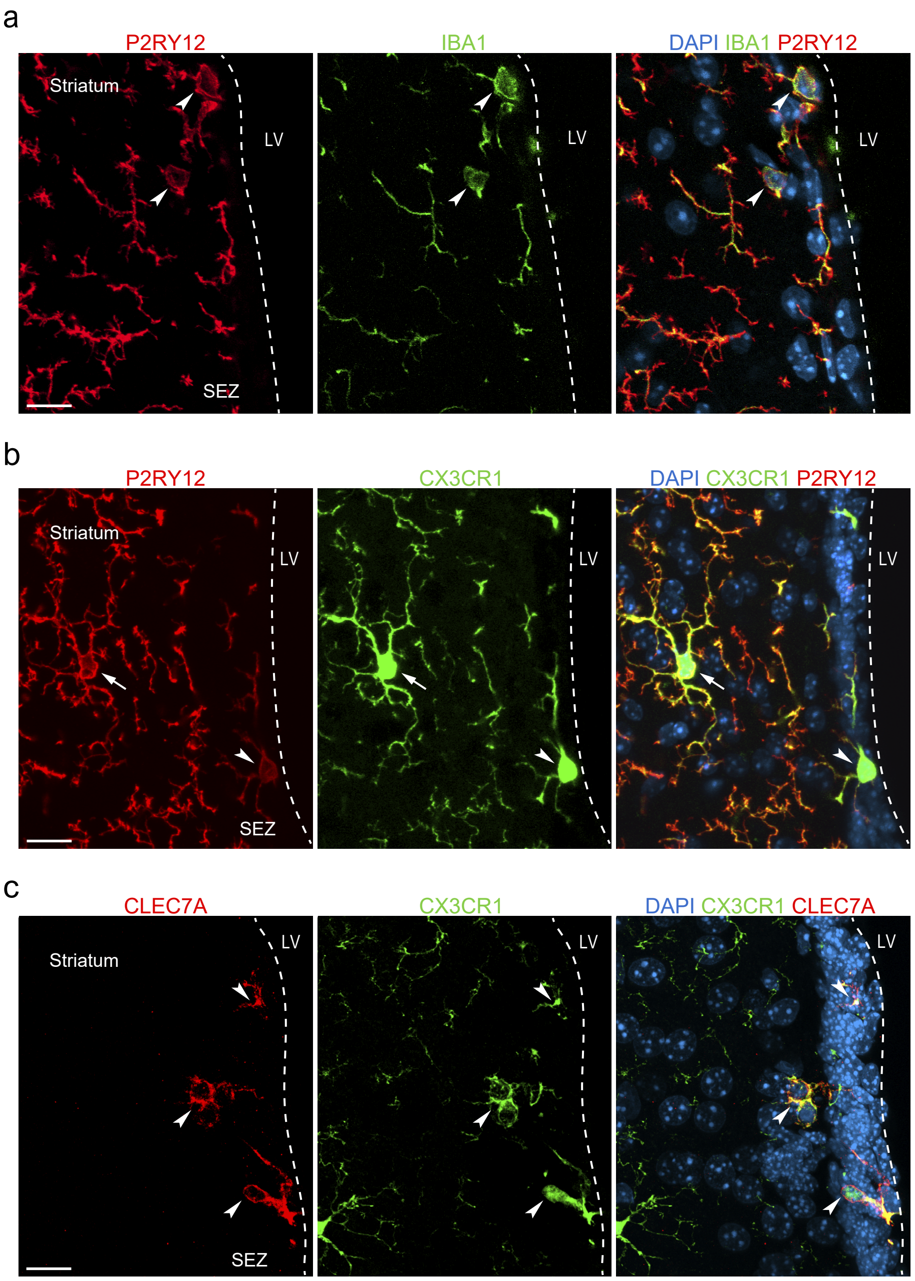

Suppl. Fig 1. ***Microglial specific stainings.*** **a.** Confocal images of coronal sections from the SEZ of 2-month-old mice, showing immunostainings for IBA1 (green) and P2YR12 (red). **b.** Confocal images of coronal sections from the SEZ of 2-month-old *Cx3cr1*$^{+/GFP}$ knockin mice showing GFP fluorescence (green, CX3CR1) and immunostaining for P2YR12 (red). **c.** Confocal images of coronal sections from the SEZ of 2-month-old *Cx3cr1*$^{+/GFP}$ knockin mice showing GFP fluorescence (green, CX3CR1) and immunostaining for CLEC7A (red). Microglial cells are indicated by arrows (at the striatum) and arrowheads (at the SEZ). DAPI: blue. Scale bar: 20 µm.

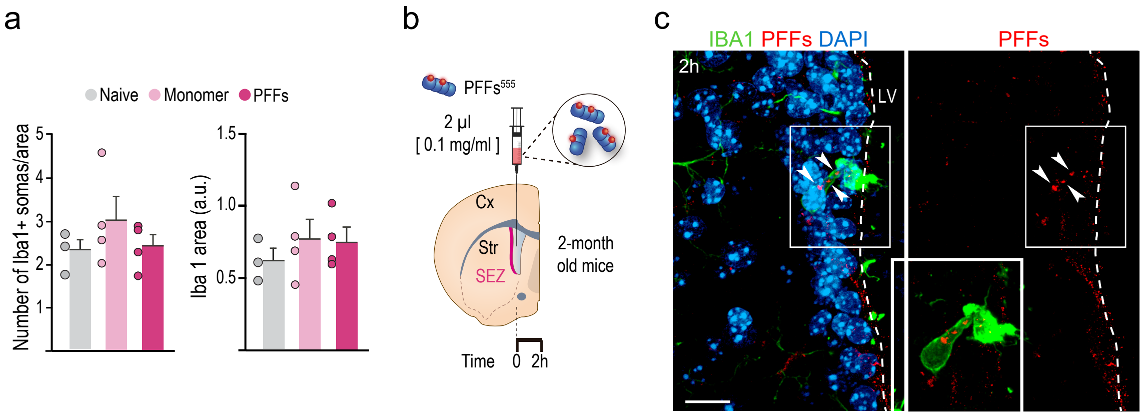

Suppl. Fig. 2. ***Microglial activation and short-term microglial uptake of αSyn PFFs.*** **a.** Quantification of the number of somas and the area occupied by microglia 15 days after the infusion of either monomeric αSyn or PFFs into the LV of 2-month-old (n = 4) or naive mice. Data are presented as mean values ± SEM. **b.** Schematic representation of the in vivo experiment. αSyn PFFs were microinjected into the right LV and the animals were euthanized two hours afterwards. **c.** Confocal images of the SEZ showing PFFs (red) and immunostaining for IBA1 (green). Arrowheads point at PFFs inside a microglial cell. DAPI: blue. Scale bar: 20 µm.

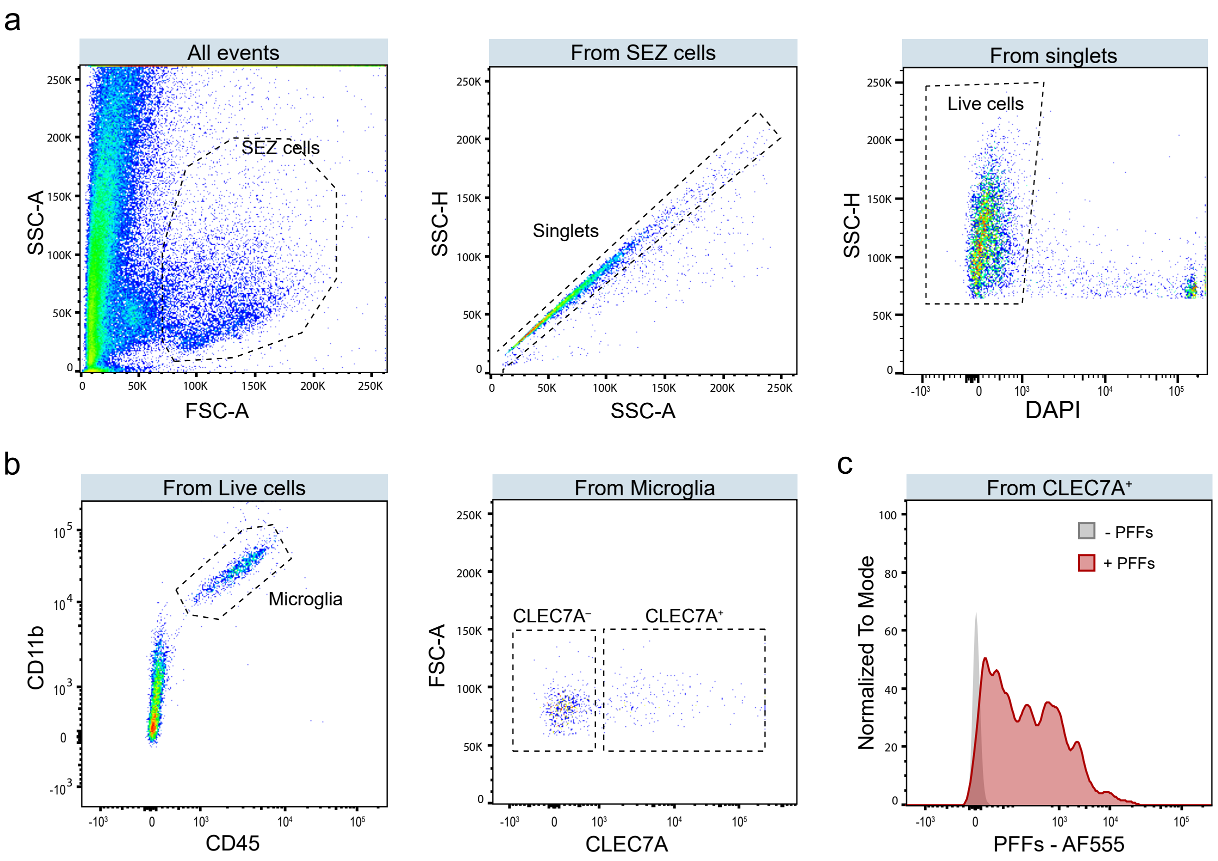

Suppl. Fig. 3. ***FACS gating strategy for microglia and ex vivo PFF phagocytosis by CLEC7A⁺ microglia*****.** **a.** Representative flow cytometry dot plots depict the initial steps of the gating strategy for live cell selection. **b.** Representative flow cytometry dot plot depicting the gating strategy for the selection of CLEC7A⁺ and CLEC7A⁻ microglia. **c.** Representative flow cytometry histogram illustrating AF555 intensity in CLEC7A⁺ microglia incubated *ex vivo* with PFFs-AF555 (red) or PBS (grey).

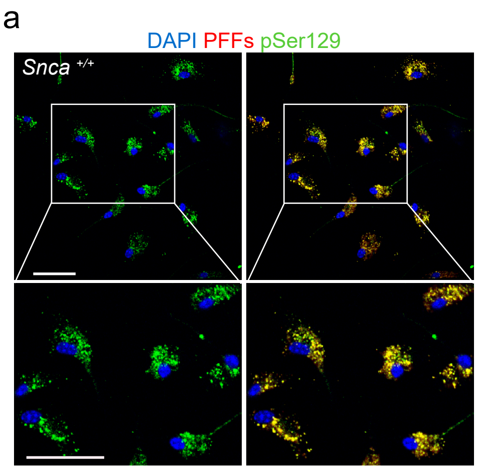

Suppl. Fig. 4. ***PFFs phosphorylation in Snca*** $^{+/+}$ ***mice.*** **a.** Detection of PFFs (red) and αSyn pSer129 (green) in *Snca* $^{+/+}$ mixed glial cultures. Scale bar: 40 μm.

a

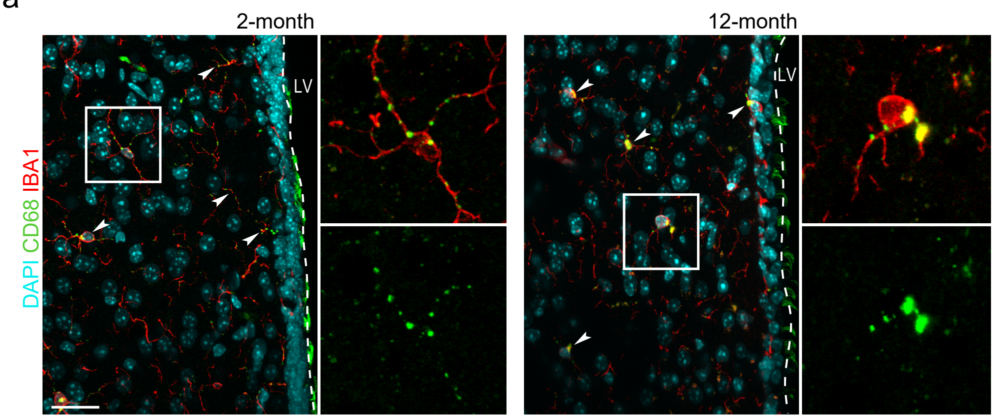

Suppl. Fig. 5. ***Age-associated lysosomal changes.*** **a.** Confocal images of the SEZ of 2- and 12-month-old mice showing immunostainings for CD68 (green) and IBA1 (red). Arrowheads point at microglial cells. DAPI: cyan. Scale bar: 30 μm.

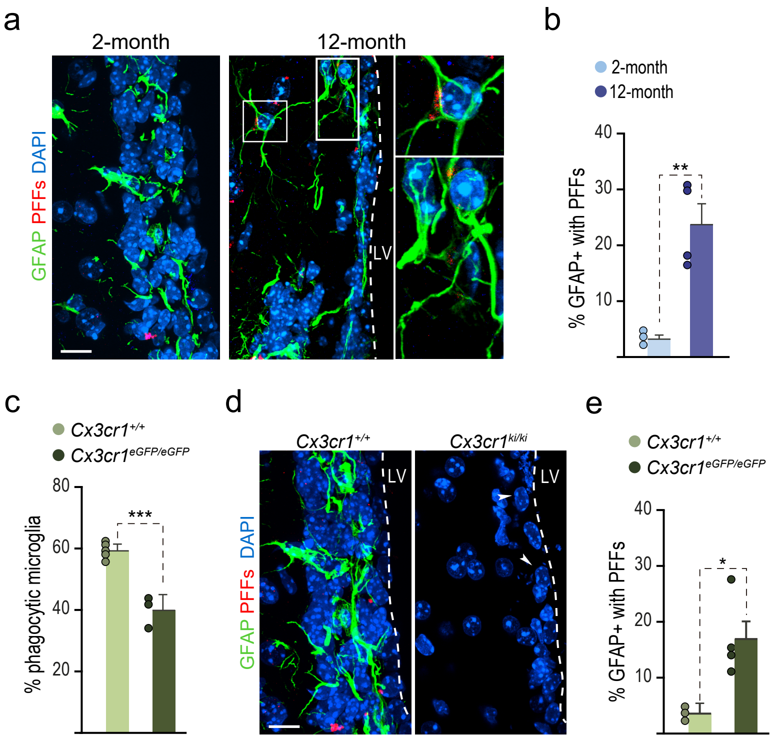

Supp. Fig. 6. Microglial impairment is associated with increased astrocytic PFF uptake. a. Confocal images of the SEZ of 2- and 12-month-old mice showing PFFs (red) and immunostaining for GFAP (green) 15 days after PFF infusion. **b.** Quantification of the percentage of astrocytes containing PFFs in 2- (n = 3) and 12-month-old (n = 4) mice. Data are presented as mean values ± SEM; Student´s t test, $p<0.01$. **c.** Flow cytometry analysis of PFF phagocytic capacity in *ex vivo* experiments with microglia isolated from the SEZ of $Cx3cr1^{+/+}$ mice (n = 5) and $Cx3cr1^{eGFP/eGFP}$ mice (n=3). Data are presented as mean values ± SEM; Student´s t-test, $p < 0.001$. **d.** Confocal images of the SEZ showing PFFs (red) and immunostaining for GFAP (green) in 2-moth-old $Cx3cr1^{+/+}$ and $Cx3cr1^{eGFP/eGFP}$ mice. **e.** Quantification of the percentage of astrocytes containing PFFs in $Cx3cr1^{+/+}$ (n = 3) and $Cx3cr1^{eGFP/eGFP}$ (n = 4). Data are presented as mean values ± SEM; Student´s t test, $p < 0.05$. DAPI: blue. Scale bar: a and d, 20 μm.

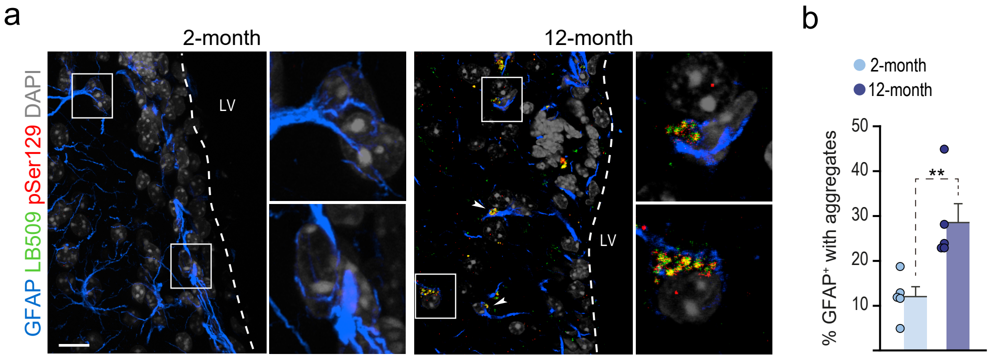

Suppl. Fig. 7. ***Increased astrocytic LB uptake in aged mice.*** **a.** Confocal images of the SEZ of 2- and 12-month-old mice showing immunostainings for human αSyn (LB509, green), pSer129 (red), and GFAP (blue) 15 days after LB-fraction infusion. Arrowheads point at astrocytes containing LB material. **b.** Quantification of the percentage of astrocytes containing LB material in 2- (n = 5) and 12-month-old (n = 5) mice. Data are presented as mean values ± SEM; Student´s t-test, $p < 0.01$. DAPI: grey. Scale bar: 20 μm.

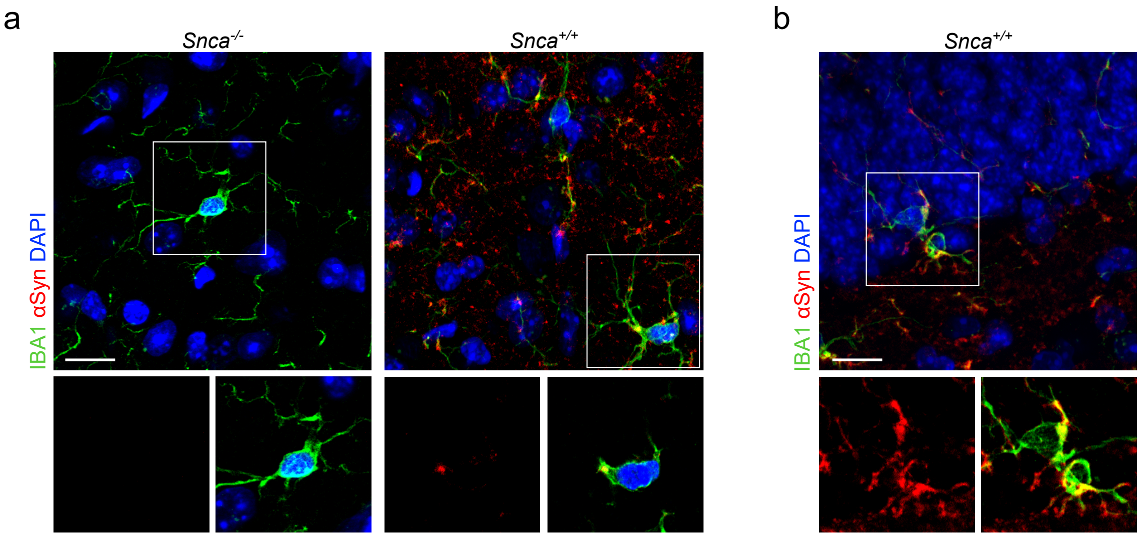

Suppl. Fig. 8. ***αSyn expression by microglia in cortex and dentate gyrus*****. a.** Confocal images of the cerebral cortex showing immunostainings for IBA1 (green) and αSyn (red). αSyn staining in *Snca*$^{-/-}$ mice is used as a control of antibody specificity. **b.** Confocal images of the dentate gyrus showing immunostainings for IBA1 (green) and αSyn (red). DAPI: blue. Scale bar: a and b, 20 μm.

Suppl. Fig. 9

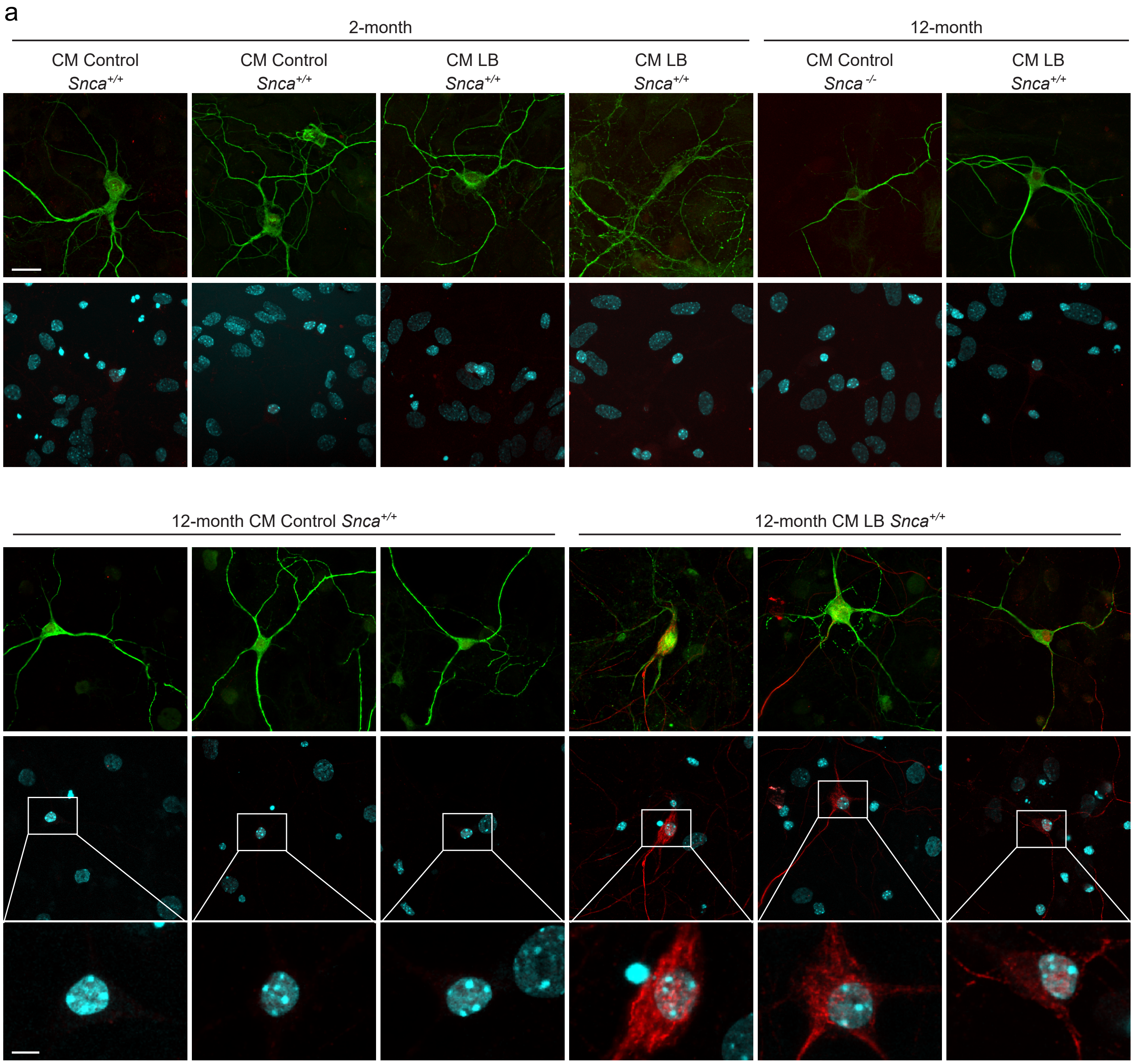

Suppl. Fig. 9. *αSyn prion-like effects induced by conditioned media obtained from microglia treated with LBs.* Representative fluorescence images of pSer129 (red) in hippocampal neuron (MAP2, green) cultures incubated with concentrated conditioned media (CM) obtained from mixed glial cells from 2-month *Snca*$^{+/+}$ mice or 12-month-old *Snca*$^{+/+}$ and *Snca*$^{-/-}$ mice treated with either PBS (control) or LBs. DAPI: cyan. Scale bars: panoramic images, 20 µm; detail images, 5 µm.

Suppl. Table 1

	Age	Gender	Other pathologies
**Controls**	65	male	Hypertension, hyperlipidemia
	74	male	L4-L5 lumbar stenosis, fibromyalgia and depression
	82	male	Hypertension, type II diabetes
**PD patients**	67	male	Hyperlipìdemia, type II diabetes
	72	male	Arthritis, depression
	80	female	Osteoporosis, depression
	84	male	Hypertension
